# A Secure Dual-Layer Fault Protection Strategy for Distribution Network with DERs: Enhancing Security in the Face of Communication Challenges

**DOI:** 10.3390/s24041057

**Published:** 2024-02-06

**Authors:** Wael Al Hanaineh, Jose Matas, Josep M. Guerrero, Mostafa Bakkar

**Affiliations:** 1 Electric Engineering Department, Polytechnic University of Catalonia (EEBE-UPC), 08019 Barcelona, Spain; jose.matas@upc.edu (J.M.); mostafa.bakkar@upc.edu (M.B.); 2 Department of Energy Technology, Aalborg University, 9200 Aalborg, Denmark; joz@et.aau.dk

**Keywords:** fault protection, total harmonic distortion, SOGI-FLL, sequence components

## Abstract

Earlier protection methods mainly focused on using communication channels to transmit trip signals between the protective devices (PDs), with no solutions provided in the case of communication failure. Therefore, this paper introduces a dual-layer protection system to ensure secure protection against fault events in the Distribution Systems (DSs), particularly in light of communication failures. The initial layer uses the Total Harmonic Distortion (THD), the estimates of the amplitude voltages, and the zero-sequence grid voltage components, functioning as a fault sensor, to formulate an adaptive algorithm based on a Finite State Machine (FSM) for the detection and isolation of faults within the grid. This layer primarily relies on communication protocols for effective coordination. A Second-Order Generalized Integrator (SOGI) expedites the derivation of the estimated variables, ensuring fast detection with minimal computational overhead. The second layer uses the behavior of the positive- and negative-sequence components of the grid voltages during fault events to locate and isolate these faults. In the event that the first layer exposes a communication failure, the second layer will automatically be activated to ensure secure protection as it operates, using the local information of the Protective devices (PDs), without the need for communication channels to transmit trip signals between the PDs. The proposed protection system has been assessed using simulations with MATLAB/Simulink and providing experimental results considering an IEEE 9-bus standard radial system. The obtained results confirm the capability of the system for identifying and isolating different types of faults, varying conditions, and modifications to the grid configuration. The results show good behavior of the initial THD-based layer, with fast time responses ranging from 6 to 8.5 ms in all the examined scenarios. In contrast, the sequence-based layer exhibits a protection time response of approximately 150 ms, making it a viable backup option in the event of a communication failure.

## 1. Introduction

In the pursuit of attaining net zero-emission goals, renewable energy sources play a crucial role. The incorporation of Distributed Energy Resources (DERs) offers numerous benefits, such as enhanced power supply reliability, increased consumer engagement, a lowered carbon footprint, and diminished greenhouse gas emissions. Nevertheless, the integration of DERs into the grid also poses challenges in terms of protection issues in the grid and coordination between the protective devices [1]. The integration of DERs transforms the radial distribution network configuration from a radial to a meshed structure, which results in the possibility of a bidirectional power flow [2]. This change to the meshed structure can lead to issues such as false tripping, the blinding of protection [3], relay over-reach, or under-reach, among other potential challenges. Furthermore, DER integration can increase fault current levels, depending on the DER type and the mode of operation with the grid [4,5,6]. Hence, this potentiality gives rise to a range of technical challenges, prominently featuring fault protection as a substantial and intricate concern [7,8]. This highlights the imperativeness for smart protective strategies to enhance the network’s security and efficiency.

In recent times, different techniques have been defined to detect and locate faults in DSs with the help of communication systems, even when there are DERs involved. Neural Network-based methods (NN) are among these approaches [9,10]. They typically assure reliable fault detection and can distinguish between different types of line faults quickly in various fault scenarios. However, they demand complex calculations, require extensive training, and may require adjustments in case of grid layout changes. They also depend on the use of communication channels.

In [11], the authors propose a two-step protection algorithm using fault-induced power changes and phase angle information for fault detection and isolation in DSs with DGs. The method effectively addresses the bi-directional power flow and ensures a rapid fault response. However, it relies on robust communication channels and has not been tested under grid reconfiguration. In [12], the authors employ negative- and positive-sequence components of the grid voltage for fault protection. However, this method has not yet been evaluated in scenarios involving inverter-based DGs.

Contrarily, recent studies have reported the importance of THD in evaluating the power grid’s quality and performance. In this regard, a number of harmonic-based fault protection methods have emerged, utilizing the harmonic content of the grid’s voltage and current for this purpose [13,14,15]. In [13], a cost-effective protection solution is presented. It employs a novel harmonic-based relay for fault detection and isolation. This relay is activated by injecting specific harmonic signals into the grid during a fault. This method serves as a directional relay, eliminating the need for a voltage transformer. However, this approach is applicable only to three-phase faults and necessitates dependable communication links. In [14], the THD and harmonics of each grid voltage phase were determined using the Fast Fourier Transform (FFT) for defining a protection method. However, implementing FFT, especially on each grid voltage line, can be computationally demanding. Additionally, this method cannot distinguish between different types of faults, relies on communication links, and exhibits slower trip activation compared to alternative methods. 

The author’s previous work [15,16] offers two THD-based techniques for fault isolation in different locations of a DS grid: one using a Second-Order Generalized Integrator structure (SOGI) [15] and another using the Multiple SOGI (MSOGI) [16]. Both approaches exhibit the fast detection of both symmetrical and unsymmetrical faults. However, a comparison between the two THD methods is provided in [15], in which the SOGI approach exhibits superior computational efficiency when implemented using a Digital Signal Processor (DSP) in terms of the number of processor cycles (c), requiring only 447 c compared to the MSOGI approach’s 1788 c. As a result, the SOGI approach was selected for ongoing, reliable fault detection.

Additionally, various protection methods employ distinct techniques such as wavelets [17,18,19], fuzzy logic [20], recursive least squares [21,22], a differential phase angle [23], s-transform [24], power spectral density and transform [25], deep belief networks [26], and Hilbert–Huang transform [27]. Nevertheless, these methodologies necessitate sophisticated computational processes and entail relatively elevated implementation expenses.

The earlier approaches mainly focused on using communication channels to transmit trip signals between PDs. This allowed for fast and coordinated fault protection. Nevertheless, these approaches came with a drawback, as they did not offer any backup plan in case of communication failure. On the other hand, some methods can operate without communication by using local measurements from the relay. These methods, though, tend to have slower fault detection times compared to the ones that rely on communication.

Therefore, to ensure fast and secure fault protection in light of potential communication failures, this paper presents a two-layer protection system for mitigating fault events in the DSs. The first layer uses the THD (${THD}_{abc}$), the estimates of amplitude voltages (${\tilde{A}}_{abc}$), and the zero-sequence components ($V_{abc0}$) of the three-phase grid voltages to develop a protection strategy performed using a Finite State Machine (FSM) for the detection and isolation of faults within the grid [15]. This layer primarily relies on communication protocols for effective coordination. The incorporation of a SOGI expedites the derivation of estimated variables and sequence components, ensuring fast detection with minimal computational overhead.

The second layer uses the behavior of the positive- and negative-sequence components of the grid voltages during fault events to locate and isolate these faults [28]. This layer operates using the local information of the PDs, avoiding the need for communication channels to transmit trip signals between the PDs. Therefore, to ensure the highest level of security and reliability in case of communication failures, a priority system is proposed between the two protection layers, thereby enhancing system redundancy. It indicates that as soon as a fault is detected, the first layer will automatically be activated and the communication signal will be verified to ensure the availability of the detection decision. In the event of a loss of communication signals, the signals are rechecked to confirm the absence of any transient issues. If the signals are received, the first layer will continue its operation to isolate the fault. In any case, if it is confirmed that communication signals are lost even after rechecking for their availability, the second layer, sequence-based fault protection, will be used as a secondary protection at each PD.

The protection system demonstrated efficient and fast fault protection, particularly in light of potential communication breakdowns. It also operates with a minimal computational load. The system was tested for various fault types, including situations with a high penetration of DG, and both low and high fault resistance. Moreover, it is adaptable for use in grid reconfiguration, providing more stable and redundant protection.

The rest sections are organized as follows: Section 2 introduces the fault detection algorithm and outlines the THD measurement. Section 3 provides the secure dual-layered fault protection mechanism. The proposed method undergoes rigorous validation through comprehensive simulations in Section 4, followed by experimental validation in Section 5. Finally, Section 6 concludes the paper findings. 

## 2. Fault Detection Algorithm

**Figure 1:**
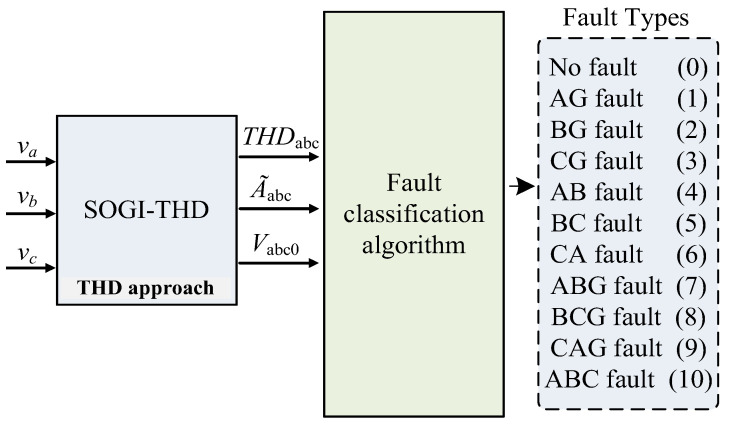
Conceptual block diagram of the THD approach for detecting faults.

In this work, each PD is equipped with an algorithm to detect faults using the measured ${THD}_{abc}$, ${\tilde{A}}_{abc}$, and $V_{abc0}$ of the grid voltage. The measured $V_{abc0}$ value is essential for fault identification in the case of phase-to-phase faults. The detection algorithm in this study offers a distinct approach for measuring the THD using the SOGI [28]. The detection additionally includes a fault classification algorithm for achieving high accuracy and detecting both symmetrical and unsymmetrical faults. Figure 1 represents the concept behind the THD method.

### 2.1. THD Measurement Method

The calculation of the THD follows the standard definition referenced in [28,29]. It is given by (1), where the sum of the squared harmonic components of a signal is divided by the square of the fundamental component, and then the square root is taken.

(1) $$THD=\frac{\sqrt{\sum_{h}{\left|A_{h}\right|}^{2}}}{A_{1}}\cdot 100,$$

where $A_{1}$ is the amplitude of the fundamental component, *h* is the harmonic order (h≠1), and $A_{h}$ is the amplitude of the *h^th^* harmonic component.

**Figure 2:**
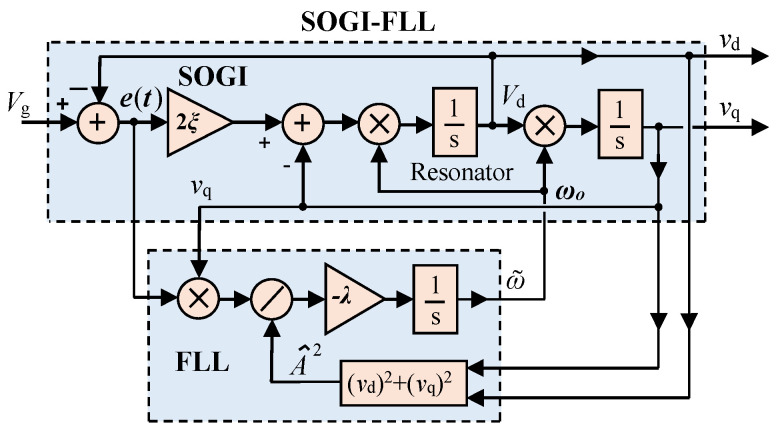
SOGI-FLL schematic diagram.

In this method, the THD calculation for each phase in the system is achieved by employing the SOGI technique, which extracts the fundamental and harmonic components [29]. Figure 2 presents the schematic of a two-coupled system, comprising a SOGI in the upper section and a Frequency-Locked Loop (FLL) in the lower section. In this figure, $v_{g}$ represents the input grid voltage, while e denotes the error. The central frequency is represented as $\omega_{o}$, and the SOGI filter’s two outputs, namely $v_{d}$ and $v_{q}$, represent the in-phase and quadrature-phase components, respectively. The FLL functions as a gradient descent estimator [30,31], delivering an estimated grid frequency denoted as $\tilde{\omega }$. The system’s damping factor is denoted as ξ, with a SOGI gain of 2ξ and an FLL gain of λ.

According to [31], $v_{d}$ and $v_{q}$ have band-pass and low-pass filter behavior, respectively, regarding the input $v_{g}$, which, assuming a constant value for $\omega_{o}$, have the following transfer functions:

(2) $$H_{d}\left(s\right)=\frac{v_{d}\left(s\right)}{v_{g}\left(s\right)}=\frac{2\xi \omega_{o}\cdot s}{s^{2}+2\xi \omega_{o}\cdot s+\omega_{o}^{2}\;\;}\;,$$

(3) $$H_{q}\left(s\right)=\frac{v_{q}\left(s\right)}{v_{g}\left(s\right)}=\frac{2\xi \omega_{o}^{2}}{s^{2}+2\xi \omega_{o}\cdot s+\omega_{o}^{2}\;\;}\;.$$

Moreover, the SOGI error signal exhibits a notch filter behavior with the following transfer function:

(4) $$H_{e}\left(s\right)=\frac{e\left(s\right)}{v_{g}\left(s\right)}=\frac{s^{2}+\omega_{o}^{2}}{s^{2}+2\xi \omega_{o}\cdot s+\omega_{o}^{2}\;\;}$$

The behavior of the FLL is mathematically represented with the following differential equation:

(5) $$\frac{d\omega }{dt}=-\frac{\lambda }{A^{2}}e{\cdot v}_{q},$$

The estimation of the grid amplitude voltage, represented as $\tilde{A}$, is achieved through the SOGI filter orthogonal outputs as follow:

(6) $$\tilde{A}\approx A=\sqrt{v_{d}^{2}+v_{q}^{2}}.$$

This method involves using a single SOGI-FLL for one of the three phases of the grid voltage and employing only a SOGI for the remaining two phases as they receive $\tilde{\omega }$ from the FLL stage of the first SOGI. Furthermore, to attain an ideal trade-off between the speed of the transient response and the capability to reject harmonic distortion, the parameter ξ is set to $1/\sqrt{2}$ [32].

Consider a single-phase grid signal that comprises fundamental and harmonic components, expressed in the following equation:

(7) $$v_{in}\left(t\right)=A_{1}\sin\left(\omega_{i}t\right)+\sum_{h}A_{h}\sin\left({h\omega }_{i}t+\varphi_{h}\right),$$

Here, $\omega_{i}$ denotes the grid frequency and $\varphi_{h}$ represents the $h^{th}$ harmonic component phase angle. The SOGI orthogonal outputs are used in this approach to derive the square of the fundamental component, $A_{1}^{2}$, as depicted in Figure 2. The characteristics of the notch filter transfer function applied to the error signal $e\left(t\right)$ in Equation (4) are utilized to calculate the remaining harmonic components, as follows:

(8) $$e\left(t\right)=\sum_{h}A_{h}\sin\left({h\omega }_{i}t+\varphi_{h}\right).$$

Subsequently, it becomes evident that by squaring $e\left(t\right)$, as in Equation (8), and applying suitable trigonometric identities, the expression for $e^{2}\left(t\right)$ can be derived as (9).

(9) $$e^{2}\left(t\right)\approx \sum_{h}\frac{A_{h}^{2}}{2}\;\left[1-\cos\left(2h\omega_{i}t+{2\varphi }_{h}\right)\right]\;\;\;\;\;\;\;\;\;\;\;\;\;\;\;+\sum_{h}\sum_{k,\;k\neq h}\frac{A_{h}\;A_{k}}{2}\;[\cos(\left(h-k\right)\omega_{i}t+\varphi_{h}-\varphi_{k})\;\;\;\;\;\;\;\;\;\;-\cos(\left(h+k\right)\omega_{i}t+\varphi_{h}+\varphi_{k})],$$

which demonstrates that the resulting DC component is precisely half of the summation of the squares of the harmonic components’ amplitudes in (9). In order to extract this DC component, a Low Pass Filter (LPF) is implemented with a fitting cut-off frequency, which leads to:

(10) $$LPF\left(e^{2}\right)=\sum_{h}\frac{A_{h}^{2}}{2}.$$

**Figure 3:**
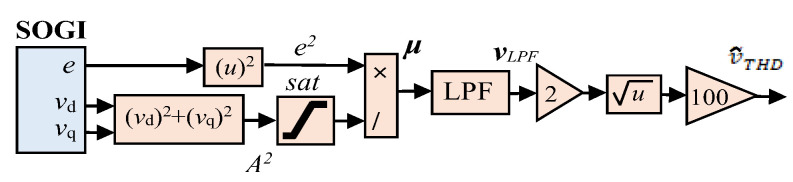
Block diagram the SOGI-THD measurement approach.

Now, using (6) and (10), scaling by a factor of two to offset the initial 1/2 gain, and applying the square root operation, the THD is achieved as:

(11) $$THD=\sqrt{\frac{2}{A^{2}}LPF\left(e^{2}\right)}\cdot 100=\sqrt{2\cdot LPF\left(\mu \right)}\cdot 100,$$

where μ is defined as $\frac{e^{2}}{A^{2}}$. Figure 3 presents the THD calculation block diagram, yielding ${\tilde{v}}_{THD}$. The multiplication by 100 is performed to express the value in a percentage scale, and the saturation function is employed to prevent division by zero. Moreover, the design of the LPF aims to achieve a balance between the transient response speed and managing distortion levels within the THD signal. 

The computation of zero-sequence components is exclusively done using the in-phase voltages from the SOGIs for each phase of the grid voltage. Specifically, $v_{da}$, $v_{db}$, and $v_{dc}$ are used in accordance with (12).

(12) $$V_{abc0}=\frac{1}{3}\left(v_{da}+v_{db}+v_{dc}\right).$$

The integration of the SOGI-FLL in this THD measurement approach offers fast fault detection. Fault occurrences are typified by abrupt and substantial fluctuations in grid voltages, eliciting responses across the entire spectrum of harmonic components. As a result, the derived THD assumes a pivotal role in expediting fault detection, constituting the foundational element of the detection algorithm.

### 2.2. Fault Classification Algorithm Stages

This algorithm employs a three-stage process to rapidly and securely detect and identify various types of faults that may arise in the grid.

#### 2.2.1. Data Acquisition Stage

During this stage, the three-phase voltages, $v_{abc}$, at each PD are measured in the time domain. Based on this measurement, the SOGI-THD approach, as explained earlier, is utilized to calculate the ${THD}_{abc}$, ${\tilde{A}}_{abc}$, and $V_{abc0}$.

#### 2.2.2. THD-LPF Fault Detection Stage

**Figure 4:**
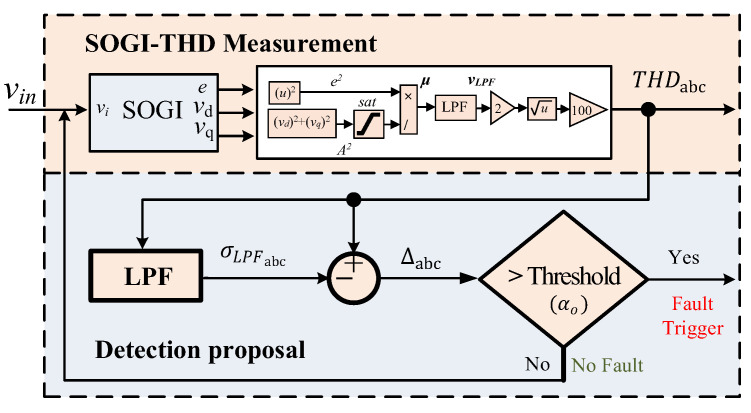
Adaptive THD-LPF fault detection block diagram using ${THD}_{abc}$ and $\sigma_{{LPF}_{abc}}$.

In this stage, an adaptive THD-LPF detection algorithm is employed to detect faults in the grid. This algorithm utilizes the ${THD}_{abc}$ and applies low-pass filtering to the THD, whose output is denoted as $\sigma_{{LPF}_{abc}}$, to enhance the reliability of fault detection, even in the presence of harmonic distortion during the fault. The design for this approach is depicted in Figure 4.

The detection process relies on the calculation of the difference between ${THD}_{abc}$ and $\sigma_{{LPF}_{abc}}$ signals at the exact moment that the fault takes place. This difference is denoted as ${∆}_{abc}$:

(13) $${∆}_{abc}={THD}_{abc}-\sigma_{{LPF}_{abc}}$$

The detection of a fault occurs when ${∆}_{abc}$ surpasses a predefined threshold, ${∆}_{abc}\geq \alpha_{o}$. In this work, the selection of the threshold $\alpha_{o}$ is designed by performing a series of simulations that encompass an array of fault scenarios and using different LPF cut-off frequencies. The primary objective is to evaluate the system’s performance, taking into account the detection speed and its robustness in the face of varying transient levels. Our findings reveal that configuring the threshold $\alpha_{o}$ at 25% provides a substantial safety margin, ensuring that the system achieves the fastest detection time during fault events while effectively preventing false detections caused by unexpected transients. This strategic approach enhances the overall reliability and robustness of the system.

**Figure 5:**
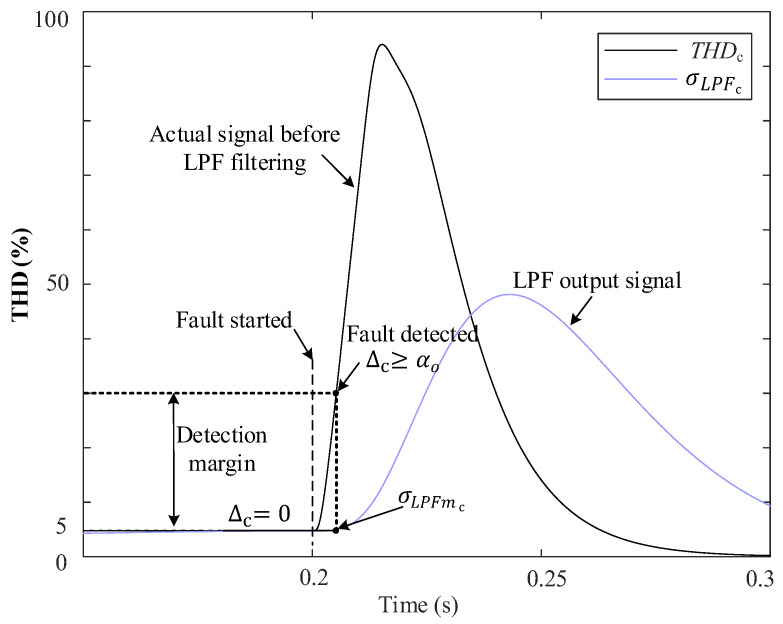
An example of ${THD}_{c}$ and $\sigma_{{LPF}_{c}}$ behavior during a fault of phase “c” using the proposed THD-LPF fault detection approach.

The low-pass filter applied to obtain $\sigma_{{LPF}_{abc}}$ has been designed with a proper cutoff frequency in order to obtain the average value of the ${THD}_{abc}$ signals. Figure 5 depicts an example of this relationship when phase “c” experiences a fault at 0.2 s. Simultaneously, a fifth harmonic with 5% amplitude distortion is present in the grid voltage before the fault. This harmonic had persisted for a suitable duration, causing the output of the low-pass filter to equal the input. As a result,$\;{∆}_{c}=0$, see Figure 5.

At the time of the fault, a rapid transient in ${THD}_{c}$ is generated, which takes time to become noticeable at the output of the low-pass filter. Furthermore, during a fault, the THD exhibits a peak-pulse waveform that exponentially decays over time, see Figure 5. However, at the precise moment of the fault, $\sigma_{{LPF}_{c}}$ remains in proximity to the THD level that was present before the fault. Therefore, the difference,$\;{∆}_{c}$, corresponds directly to the disparity between the actual THD and the previous THD level that existed in the grid at the time of the fault.

Hence, employing the low-pass filter in this manner enables the detection of abrupt rises in THD caused by faults, irrespective of the pre-existing harmonic distortion levels in the grid voltage. Once a fault is detected, the algorithm reads and saves the value of $\sigma_{{LPF}_{abc}}$, denoted as $\sigma_{{LPFm}_{abc}}$, which will be utilized in subsequent stages of the identification process.

#### 2.2.3. Fault Identification

In this stage, two thresholds are utilized for the fault identification process. The first threshold, $\sigma_{{LPFm}_{abc}}$, is used for comparing with ${THD}_{abc}$. When a fault is detected, it is checked if ${THD}_{abc}>\sigma_{{LPFm}_{abc}}$. Moreover, a tolerance range of 7.5% for the grid voltage drop at the medium voltage level is set as the second threshold, denoted as Δv. This threshold is used to compare with ${\tilde{A}}_{abc}$ during the fault. The selection of this value aligns with the technical requirements specified in the Spanish grid code for ensuring reliable energy integration [33].

**Figure 6:**
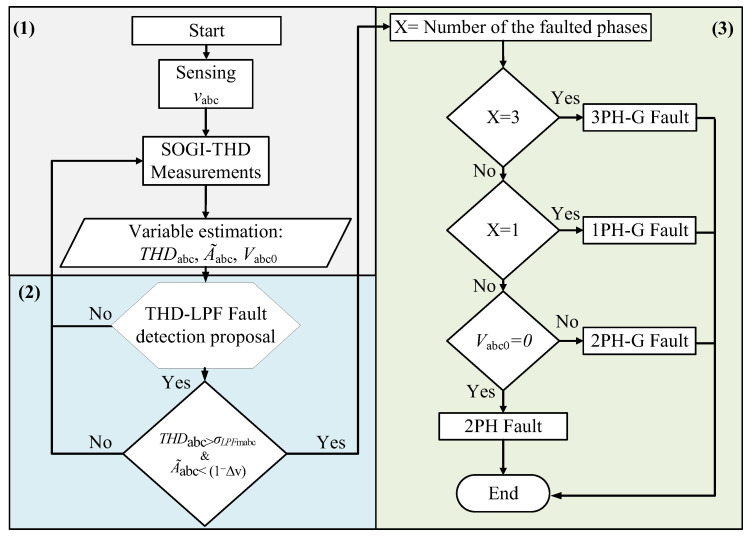
Scheme of the fault classification algorithm.

During a fault event, the voltage drop in the faulted phases leads to a sudden decrease in ${\tilde{A}}_{abc}$. As a result, the estimated value of the faulted phase falls below the threshold, specifically ${\tilde{A}}_{abc}<\left(1-\Delta v\right)$ pu. Then, achieving these conditions is considered as an indication of a fault event. Therefore, at this level, $V_{abc0}$ is employed to distinguish between a phase-to-phase (2PH) fault and a phase-to-phase-ground (2PH-G) fault, as their initial conditions are identical. For a 2PH fault, $V_{abc0}=0$, whereas $V_{abc0}\neq 0$ in the case of a 2PH-G fault [34]. A total of 11 fault categories, numbered from 0 (no fault) to 10 (three-phase fault), have been used to adopt a decision and to classify the faults, as shown in Figure 1. Figure 6 outlines the structural framework of the fault classification algorithm.

## 3. Secure Dual-Layered Fault Protection Mechanism

A secure protection is proposed using a dual-layer protection strategy. The first layer consists of a THD-based Finite State Machine (FSM), which works once the fault is detected by the SOGI-THD detection algorithm and using the measured ${THD}_{abc}$, ${\tilde{A}}_{abc}$, and $V_{abc0}$ of the PDs of each DL. Each DL has an FSM that will be coordinated with the others in the PDs by means of the communication for secure protection. The second layer is a sequence-based fault location algorithm that will use the sequence-voltage components’ behavior during the fault to isolate it. This algorithm is employed at each PD without the need for communication links; therefore, this level can be used as pack-up protection in the case of poor communication to provide reliable and redundant protection.

### 3.1. Proposed System

**Figure 7:**
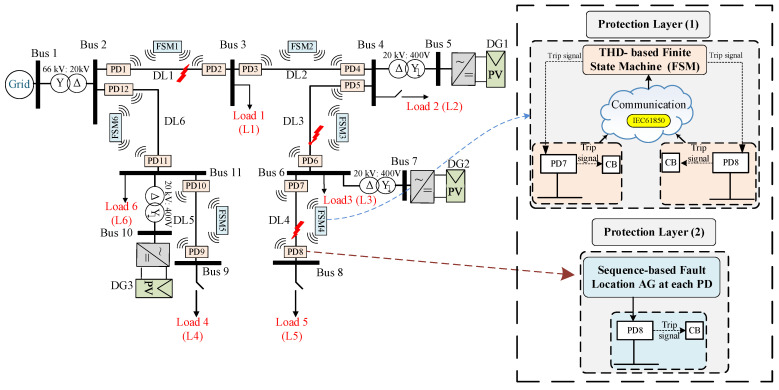
A single-line diagram of the proposed IEEE 9-bus system with the two protection layers.

The fault protection methodology presented in this study can be implemented in various DSs. In order to validate the effectiveness of the proposed approach, the IEEE 9-bus standard [35] radial system with DGs was selected as the test case, as shown in Figure 7. The system comprises a main grid and multiple DGs connected via various buses. The high-voltage (HV) grid operates at a rated voltage of 66 kV and a power rating of 25 MVA. The HV grid is connected to six distribution lines (DLs), labeled DL1 to DL6, via a step-down transformer. The HV/MV transformer configuration is a star/delta (YNd11). Each DL consists of two PDs positioned on either side of the line and an FSM. Inside each PD, there is a fault detection relay that operates in coordination with the FSM as the primary protection layer to isolate the line during fault events. Communication channels facilitate the exchange of signals between the PDs and the FSM, as well as between the FSMs themselves, to ensure the coordinated tripping of the faulted DLs. Additionally, in case of the communication failure, each PD employs a sequence-based algorithm as a secondary protection layer. Local loads (L1 to L5) are connected at the terminations of each DL. Three DGs, identified as DG1 to DG3, are linked to different buses via an MV/LV transformer. All loads and DGs are connected to the low-voltage side of the system through a delta/star grounded configuration (Dyn11) [36]. The system parameters, including the voltage ratings, power ratings, transformer configurations, and other relevant details, are provided in Table 1.

It is important to mention that the integration of DGs into this specific DS configuration creates multiple pathways for delivering electric power to customers. This enhances system reliability and grid efficiency. However, it introduces the possibility of a bidirectional power flow, which adds complexity to the fault protection process. Furthermore, protection techniques often rely on communication-based coordination, potentially creating a vulnerability if communication fails. Hence, new protection strategies are needed to ensure system safety in scenarios with a bidirectional power flow and potential communication failures. Consequently, the proposed approach will undergo a thorough examination and testing under different scenarios. This strategy aims to ensure reliable system operation, providing effective protection regardless of the power flow direction or communication status.

### 3.2. THD-Based Finite State Machine

**Figure 8:**
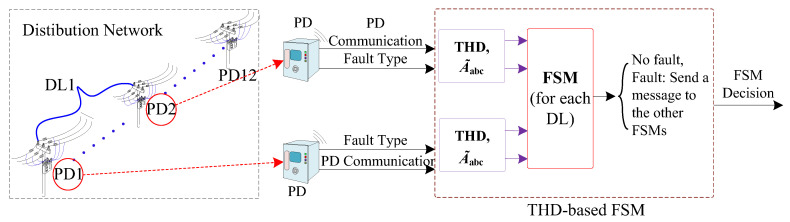
Conceptual block diagram of the THD-based FSM protection layer.

**Figure 9:**
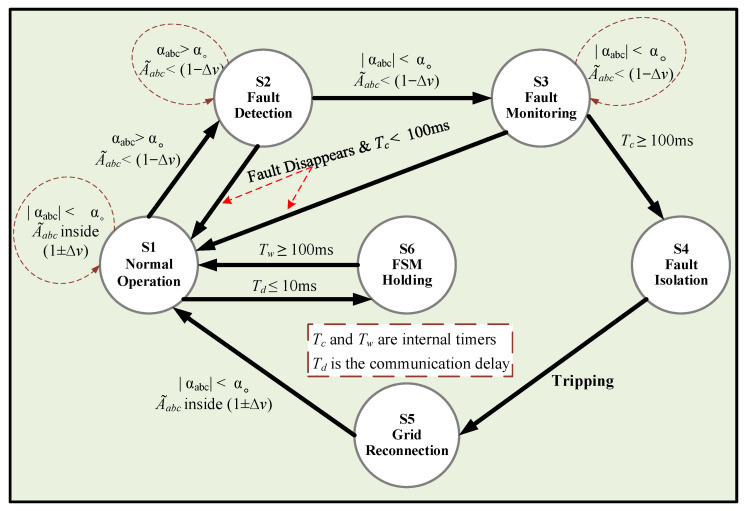
State flow chart diagram of an FSM.

In this work, the FSM described in [15] is utilized as an initial layer of protection, which relies on two distinct communication protocols (see Figure 7): a Wi-Fi protocol between the PDs and FSM [37] and the IEC 61850 protocol [38] between the FSMs themselves for proper coordination and to ensure effective protection. Figure 8 depicts the conceptual diagram of this protection layer. In Figure 7, six FSMs are designated for detecting and isolating the faults in various locations of the DS. Each FSM in the system is allocated to a DL with two PDs and comprises six unique states, whose state flow chart diagram is shown in Figure 9.

At normal operation, the system functions around the nominal values during state S1 (Normal Operation), which means that the FSM remains in S1 on standby, waiting for any faults to occur. Whenever a fault occurs, the PD closest to the fault is the quickest to detect it. Therefore, upon detection, as explained in Section 2.2.2, the PD sends a detection message to the FSM, enabling it to initiate its process and transition to state S2 (Fault Detection). It is worth noting that the delay in transmitting a detection message using the Wi-Fi protocol is negligible, taking less than 1 ms [39].

As a result, the FSM of a faulted DL is the nearest FSM to the fault; therefore, it is always able to detect the fault more rapidly compared to the other FSMs. This fact is used as a kernel for developing a fast and efficient fault isolation approach.

When the FSM of the faulted DL moves into state S2, a timer called $T_{c}$ begins running to track the fault duration. Additionally, a new fault signal is dispatched to the remaining FSMs, informing them of the event and enabling them to stop their processes. The transmission delay for this fault message, referred to as $T_{d}$, is estimated to be approximately 10 ms [38].

Then, when the peak in the THDs eventually disappears, the FSM moves to state S3 (Fault Monitoring), where it systematically monitors the fault with the intention of isolating only permanent faults and avoiding undesirable tripping. It is worth noting that temporary faults are typically resolved within 100 ms without requiring any protective action [40,41]. Therefore, during state S3, the timer $T_{c}$ keeps running, and once it reaches the maximum limit of 100 ms, the FSM moves to state S4 (Fault Isolation), which is intended for tripping the nearest circuit breaker and isolating the fault.

Meanwhile, when the FSM is in S2 or S3, if the fault disappears, as in a temporary fault, the system returns to operate within the normal values. Therefore, the FSM returns to S1 (Normal Operation). In state S4, after fault isolation, the FSM resets $T_{c}$ to zero and transitions to state S5 (Grid Reconnection), where it waits for the reconnection of the line by the grid operator or maintenance crew to restore the FSM to its normal operating state (S1).

It is worth noting that if a non-faulted FSM is in S1 and a fault signal is received from a faulted FSM, the non-faulted FSM moves to S6 (Holding FSM) to wait for the fault to be resolved or isolated. During this time, a timer named $T_{w}$ starts counting up, and when it reaches 100 ms, the FSM returns to its normal operating state (S1).

### 3.3. Sequence-Based Fault Location Algorithm

**Figure 10:**
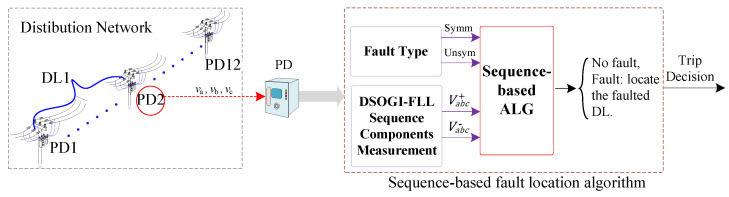
Conceptual block diagram of the sequence-based protection layer.

**Figure 11:**
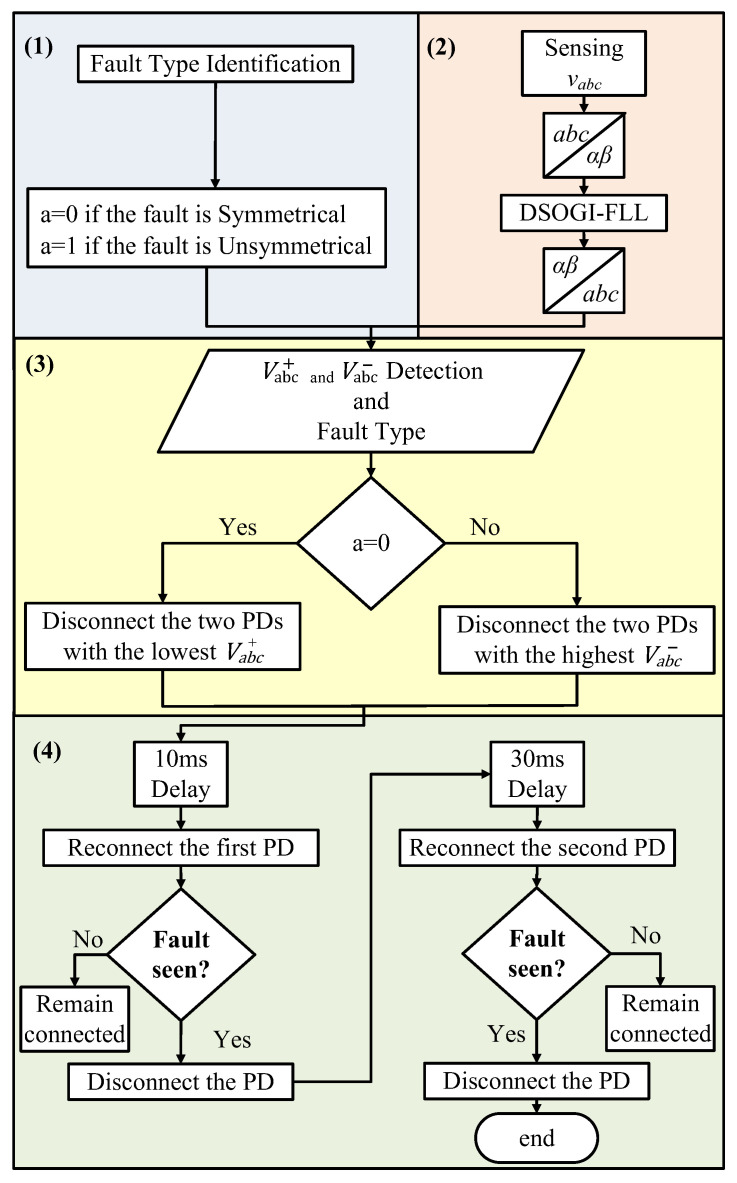
Block diagram of the sequence-based algorithm flow chart.

The fault location algorithm is a secondary layer in the system that operates at each PD and employs the voltage sequence components of the PD during fault events [28,42] to determine the fault’s location. The algorithm’s kernel is based on the behavior of the sequence components, which depends on the type of fault, whether it is symmetrical or unsymmetrical, and has the advantage of not requiring communication links between PDs. Moreover, this algorithm relies only on local information gathered by each PD to detect the faulted DL. Figure 10 depicts the conceptual diagram of this protection layer. The algorithm is composed of four stages, which are further elaborated below and depicted in Figure 11.

#### 3.3.1. Fault Identification

The fault identification involves identifying the fault type using the procedure illustrated in Figure 6, where faults are categorized into two primary types: symmetrical faults (3PH-G) and unsymmetrical faults (1PH-G, 2PH-G, and 2PH).

#### 3.3.2. Sequence Components Detection

At this stage, a dual-SOGI (DSOGI) structure [30] is used to extract the sequence components of the three-phase voltage. This operation is carried out individually for the α  and β omponents subsequent to the application of a waveform transformation. The resulting sequence components within the αβ  frames are defined in Equations (14) and (15), where $v_{\alpha }^{+}$ and $v_{\beta }^{+}$ denote the positive sequences within the αβ frames, while $v_{\alpha }^{-}$ and $v_{\beta }^{-}$ represent the corresponding negative sequences.

(14) $${v_{\alpha }=v}_{\alpha }^{+}+v_{\alpha }^{-}$$

(15) $${v_{\beta }=v}_{\beta }^{+}+v_{\beta }^{-}$$

The derivation of the positive and negative sequence components of the three-phase voltage is done by the means of the following equations based on Lyon’s methods [43,44]:

(16) $$\begin{array}{l} V_{abc}^{+}=\left[\begin{array}{c} T_{+} \end{array}\right]\;V_{abc}\\ \left[\begin{array}{c} v_{a}^{\left(+\right)}\\ v_{b}^{\left(+\right)}\\ v_{c}^{\left(+\right)} \end{array}\right]=\frac{1}{3}\left[\begin{array}{ccc} 1 & a & a^{2}\\ a^{2} & 1 & a\\ a & a^{2} & 1 \end{array}\right]\left[\begin{array}{c} v_{a}\\ v_{b}\\ v_{c} \end{array}\right] \end{array},$$

(17) $$\begin{array}{l} V_{abc}^{-}=\left[\begin{array}{c} T_{-} \end{array}\right]\;V_{abc}\\ \left[\begin{array}{c} v_{a}^{\left(-\right)}\\ v_{b}^{\left(-\right)}\\ v_{c}^{\left(-\right)} \end{array}\right]=\frac{1}{3}\left[\begin{array}{ccc} 1 & a^{2} & a\\ a & 1 & a^{2}\\ a^{2} & a & 1 \end{array}\right]\left[\begin{array}{c} v_{a}\\ v_{b}\\ v_{c} \end{array}\right] \end{array},$$

Here, the parameter $a=1∠120^{0}$ denotes the Fortescue phase-shifting operator. The grid voltage undergoes a conversion from the abc to the αβ  frames, a process executed through the application of the *Clarke* transformation, as in Equation (18).

(18) $$\begin{array}{l} V_{\alpha \beta }=\left[\begin{array}{c} T_{\alpha \beta } \end{array}\right]{\;V}_{abc}\\ \left[\begin{array}{c} v_{\alpha }\\ v_{\beta } \end{array}\right]=\sqrt{\frac{2}{3}}\left[\begin{array}{ccc} 1 & -\frac{1}{2} & -\frac{1}{2}\\ 0 & \frac{\sqrt{3}}{2} & -\frac{\sqrt{3}}{2} \end{array}\right]\left[\begin{array}{c} v_{a}\\ v_{b}\\ v_{c} \end{array}\right] \end{array}.$$

In light of this, Equations (19) and (20) are used for the computation of the positive and negative-sequence voltage components in the αβ frames. This equation uses a 90°-lagging phase-shifting operator in the time domain, resulting in an in-quadrature depiction of the input waveforms.

(19) $$\begin{array}{l} V_{\alpha \beta }^{+}=\left[\begin{array}{c} T_{\alpha \beta } \end{array}\right]V_{abc}^{+}=\left[\begin{array}{c} T_{\alpha \beta } \end{array}\right]\left[\begin{array}{c} T_{+} \end{array}\right]V_{abc}\\ \left[\begin{array}{c} v_{\alpha }^{\left(+\right)}\\ v_{\beta }^{\left(+\right)} \end{array}\right]=\left[\begin{array}{c} T_{\alpha \beta } \end{array}\right]\left[\begin{array}{c} T_{+} \end{array}\right]{{\left[\begin{array}{c} T_{\alpha \beta } \end{array}\right]}^{T}V}_{\alpha \beta }=\frac{1}{2}\left[\begin{array}{cc} 1 & -e^{-j\left(\frac{\pi }{2}\right)}\\ e^{-j\left(\frac{\pi }{2}\right)} & 1 \end{array}\right]\left[\begin{array}{c} v_{\alpha }\\ v_{\beta } \end{array}\right] \end{array},$$

(20) $$\begin{array}{l} V_{\alpha \beta }^{-}=\left[\begin{array}{c} T_{\alpha \beta } \end{array}\right]V_{abc}^{-}=\left[\begin{array}{c} T_{\alpha \beta } \end{array}\right]\left[\begin{array}{c} T_{-} \end{array}\right]V_{abc}\\ \left[\begin{array}{c} v_{\alpha }^{\left(-\right)}\\ v_{\beta }^{\left(-\right)} \end{array}\right]=\left[\begin{array}{c} T_{\alpha \beta } \end{array}\right]\left[\begin{array}{c} T_{-} \end{array}\right]{{\left[\begin{array}{c} T_{\alpha \beta } \end{array}\right]}^{T}V}_{\alpha \beta }=\frac{1}{2}\left[\begin{array}{cc} 1 & e^{-j\left(\frac{\pi }{2}\right)}\\ -e^{-j\left(\frac{\pi }{2}\right)} & 1 \end{array}\right]\left[\begin{array}{c} v_{\alpha }\\ v_{\beta } \end{array}\right] \end{array},$$

**Figure 12:**
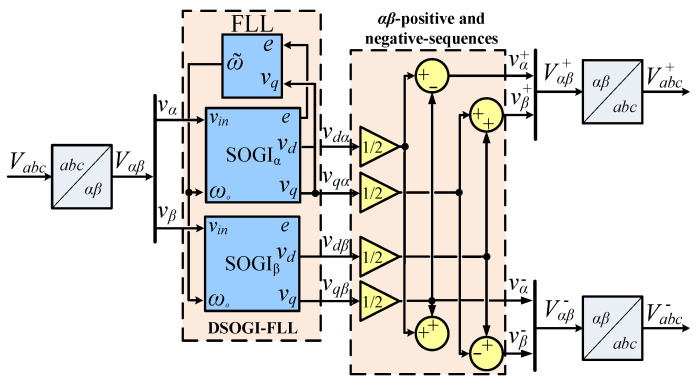
The detection block diagram of the positive- and negative-sequence component using the DSOGI-FLL approach.

In Figure 12, the DSOGI-FLL block diagram is depicted, showcasing its functionality for obtaining the positive- and negative-sequence components of a three-phase voltage at a frequency of $\omega_{\circ }$. This configuration incorporates two SOGIs operating on the αβ  frame to yield input signals for the sequence voltages calculation block. Within this block, the transformations in Equations (19) and (20) are applied with precision to compute the positive- and negative-sequence components.

#### 3.3.3. PDs’ Initial Disconnection

The behavior of the sequence voltages is dependent on the type of fault detected, which is the kernel of this algorithm for the secure location of the faulted DL. For instance, in the case of symmetrical faults, i.e., a 3PH-G fault at the end of DL3, see Figure 7, the faulted DL can be located by monitoring the Positive-Sequence Voltage (PSV) during the fault. The simulations showed that during the fault, the PSV at PD6 of DL3, which is the nearest to the fault, will be lower than that of the other unfaulted DLs [28,42]. As a result, PD6 with the lowest PSV value will be disconnected to isolate the faulted DL more quickly than the other PDs.

In the case of unsymmetrical faults, i.e., a 1PH-G fault at the end of DL1, see Figure 7. The location of the faulted DL can be determined by examining the Negative-Sequence Voltage (NSV) behavior. Specifically, the NSV at PD2 of DL1 will be higher than that of any other PDs. Thus, in this scenario, PD2 that detects the fault with the highest negative-sequence value will trip first, before the other PDs.

**Figure 13:**
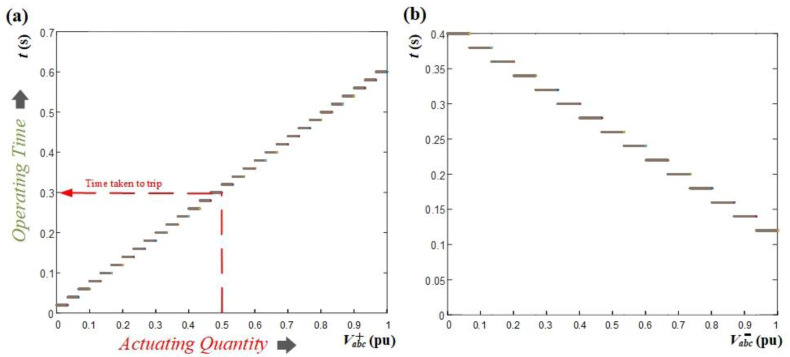
Definite time curve: (**a**) Positive-sequence voltage (**b**) Negative-sequence voltage.

It is crucial to emphasize that two PDs located on opposite sides of each bus are connected, i.e., “bus 6 is connected to both PD6 and PD7”, see Figure 7. This implies that at each bus, two points with identical voltages are acquired, resulting in matching sequence voltages. Consequently, if a fault happens, both PDs will be disconnected simultaneously as an initial protection measure. Therefore, the disconnection of the aforementioned PDs conforms to the definite time-voltage characteristic curves, as illustrated in Figure 13, and is predicated upon the type of fault. 

These curves demonstrate the correlation between the positive- and negative-sequence voltages and the corresponding time taken by each PD to activate. The design of these curves ensures that the PD transmits a trip signal in accordance with the measured sequence voltage value. These curves are specifically formulated to comply with IEEE time standards [45,46] and are compatible with various electrical networks. The characteristic curves have undergone a meticulous design process and have been refined through numerous fault simulations preformed at different locations within the system. The curves have been continuously updated to ensure their applicability for all possible fault cases at different locations in the system. However, the DLs are considered relatively short with low impedance, resulting in only slight voltage differences between both ends of the line. Therefore, to ensure effective coordination, the curves have been crafted to be highly sensitive to even small changes in sequence voltages. For example, in the case of the positive-sequence voltage curve, the trip time is precisely adjusted by 0.02 s when the positive-sequence voltage changes by 0.0333 pu. Similarly, for the negative-sequence voltage curve, the time is adjusted by 0.02 s with a change of 0.0667 pu in the negative-sequence voltage. These specific values have been thoughtfully selected based on the system sequence voltages obtained from extensive fault simulations conducted at various system locations to prevent any potential miscoordination issues. Furthermore, as both ends of the distribution line need to be disconnected during a fault, the proposed fault location algorithm can effectively operate using the concept of PD reconnection, ensuring a reliable and robust protective system.

**Figure 14:**
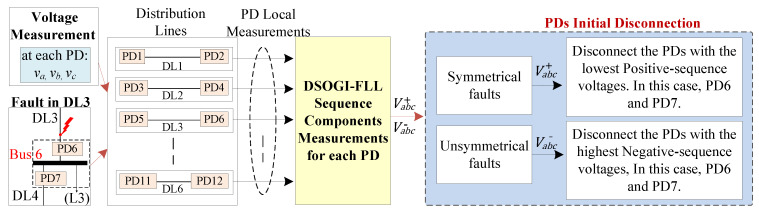
Conceptual block diagram of PDs’ initial disconnection stage of the sequence-based fault location algorithm, an example when a fault event occurs in DL3.

Figure 13a depicts the time curve for the PSV magnitude. This curve is employed in scenarios of symmetrical faults, wherein the lowest PSV triggers the fastest trip. In contrast, Figure 13b illustrates the time curve for the NSV magnitude. This curve is used in the case of unsymmetrical faults, where the highest NSV is the first to trigger the trip. The conceptual block diagram of the PD’s first disconnection stage in case of a fault at DL3 is given in Figure 14.

#### 3.3.4. PDs’ Re-Energization

As previously mentioned, as a first protection action, the PDs located on both sides of the bus connected to a faulted DL will be disconnected. However, it is only necessary to disconnect the PD on the faulty side, while the other PD, on the unfaulty side, should remain connected. To address this issue, the algorithm has been developed to sequentially reconnect each PD with a specific delay and check for fault existence. As a result, the PD that continues to detect the fault will be permanently disconnected, while the other will remain connected. There are two possible situations for reconnecting the PDs at the faulted DL, depending on the location of the fault.

(1) *The fault at any end of the DL*

**Figure 15:**
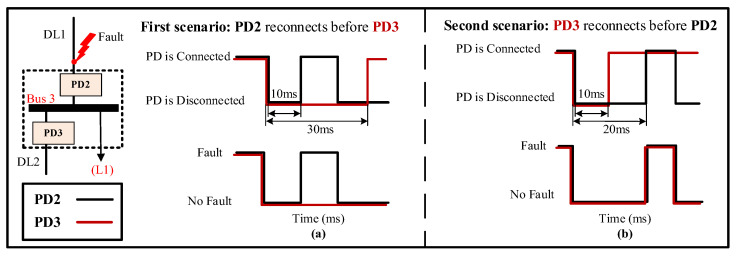
PDs’ re-energization scenarios in case of a fault at the end of the line. (**a**) First scenario. (**b**) Second scenario.

In this case, two scenarios for the reconnection of the PDs at the faulted DL are possible. For instance, if there is a fault in DL1, both PD2 and PD3, which are connected to bus 3, will trip simultaneously. Following this, the reconnection scenarios will be implemented. The first scenario involves the faster reconnection of PD2 compared to PD3, while the second scenario involves the faster reconnection of PD3 compared to PD2. Figure 15 provides an illustration of these two scenarios.

The first one, Figure 15a, involves the reconnection of PD2 first, after a specified delay, which can range from a few milliseconds to several seconds [47]. In this work, the delay is set to 10 ms [48]. As a result, PD2 will detect the fault once again and be permanently disconnected. On the other hand, PD3 will be reconnected after a longer delay of 30 ms [48] to ensure complete fault isolation on the first side of DL1. As a result, PD3 will remain connected since it does not detect the fault.

The second one, Figure 15b, involves the reconnection of PD3 first, after a delay of 10 ms. Given that the fault occurred at DL1 and PD2 remains disconnected, PD3 does not detect the fault and thus remains connected. Following this, PD2 is reconnected after a delay of 20 ms [48]. Nonetheless, due to the persisting presence of the fault, PD2 identifies the fault once again, resulting in its permanent disconnection.

Additionally, to achieve complete fault isolation at DL1, it is essential to disconnect the second end of the line as well, see in Figure 7. Therefore, after disconnecting the first end of DL1 and re-measuring the sequence voltages of the grid, it is observed that the second end of DL1 displays the lowest PSVs during symmetrical faults and the highest negative-sequence voltages during unsymmetrical faults, which aligns with the algorithm’s core principles. Thus, the same technique employed for the first end is used at the second end to ensure reliable protection. Note that a comprehensive discussion of these findings is given in the results section.

(2) *The fault at the middle of the DL*

**Figure 16:**
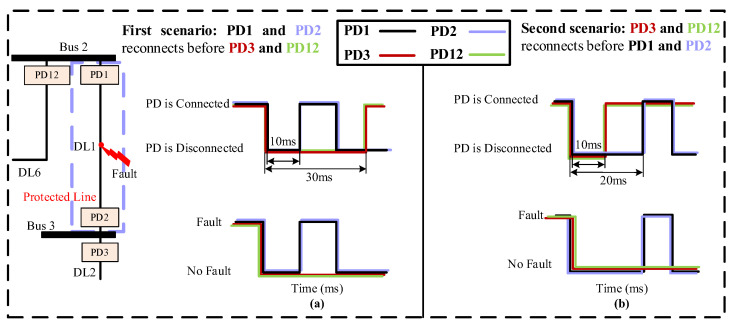
PDs’ re-energization scenarios in case of a fault in the middle of the line. (**a**) First scenario. (**b**) Second scenario.

If a fault occurs in the middle of the DL, as depicted in Figure 16, the four PDs connected to the two buses at the ends of the faulted DL will share the same sequence voltages. In this case, the reconnection technique will be employed at both ends of the DL. For instance, when a fault happens in the middle of DL1, then PD1, PD2, PD3, and PD12 will be disconnected simultaneously due to their identical sequence voltages. Following this, two reconnection scenarios will be implemented. 

The first one, Figure 16a, involves the faster reconnection of PD1 and PD2 compared to PD3 and PD12. Therefore, PD1 and PD2 are reconnected first, after a specified delay of 10 ms. As a result, PD1 and PD2 will detect the fault once again and be permanently disconnected. On the other hand, PD3 and PD12 will be reconnected after a longer delay of 30 ms to ensure the complete isolation of DL1. As a result, PD3 and PD12 will remain connected since they do not detect the fault.

The second one, Figure 16b, involves the faster reconnection of PD3 and PD12 compared to PD1 and PD2. Therefore, PD3 and PD12 are connected first, after a delay of 10 ms. Given that the fault occurred at DL1 and PD1 and PD2 remain disconnected, PD3 and PD12 do not detect the fault and thus remain connected. Following this, PD1 and PD2 are reconnected after a delay of 20 ms. Nonetheless, due to the persisting presence of the fault, PD1 and PD2 identify the fault once again, resulting in its permanent disconnection.

### 3.4. Priority System

To ensure the highest level of security and reliability in case of communication failures, a priority system is proposed between the two protection levels, thereby enhancing system redundancy.

**Figure 17:**
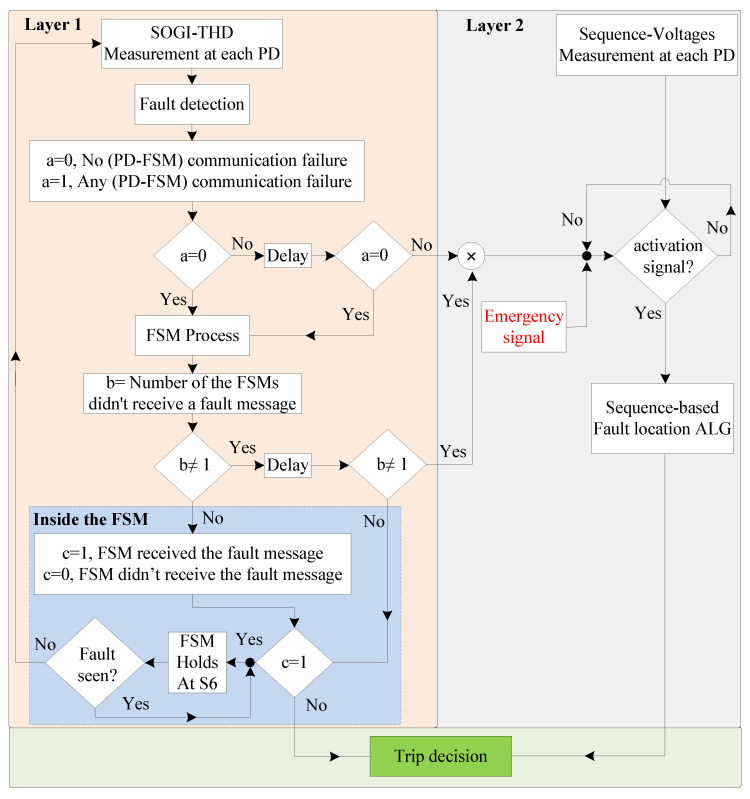
The diagram of the two-layered priority system.

The flow chart of the two-layered priority system is illustrated in Figure 17, which is critical for maintaining the system’s integrity. It indicates that as soon as a fault is detected at the PD using the SOGI-THD, a detection message is immediately transmitted to the FSM via Wi-Fi communication links. Thus, before the FSM is initiated, the communication signal is verified to ensure the availability of the detection decision. This verification involves checking if the message received at the FSM receiver is (1), indicating a successful communication, or (0), signifying message loss. In case of the failure of the PDs at a faulted DL (resulting in the loss of communication signals), a delay of 20 ms [48] is introduced, and then the signals are rechecked to confirm the absence of any transient issue. If the signals are received, the FSM begins its operation, as explained in Figure 9.

As stated earlier, the FSM of the faulted DL is the quickest to detect the fault and sends a fault signal to the other FSMs via communication links for coordinated protection. Therefore, to prevent false tripping, this signal is verified at all FSMs. With six FSMs in total, each corresponding to a DL, under normal conditions, five FSMs should receive the communication message (indicated by a signal of 1 at each receiver), while the remaining FSM, responsible for transmitting the messages to the others, registers a signal of 0 at its receiver. Now, following Figure 17, if only one FSM does not receive the message, it indicates that there is no communication failure, which is expected to be the FSM of the faulted DL sending the message to the other FSMs. However, if there is a communication failure (i.e., at least one signal is lost), another 30 ms delay is introduced [48], and then the signals are rechecked to confirm the failure.

Nevertheless, if the fault message is received, each FSM will independently check whether it has received the message. If so, the FSM will move to S6 (FSM Holding) to stop its operation, indicating that it is not faulted. Otherwise, it will continue its operation and make a trip decision (Layer 1). Once on hold at S6, the FSM will continuously monitor for fault clearance before returning to normal operation.

In any case, if it is confirmed that communication signals are lost even after rechecking for their availability, the sequence-based fault location algorithm (see Figure 11) will be used as a secondary protection at each PD. This second layer of protection can be activated using two signals, namely the Activation signal and the Emergency signal. The system automatically generates an activation signal for the second layer at each PD if the signals in the first layer cannot be received due to either communication problems or vulnerability. Additionally, the emergency signal can manually activate the second layer of protection at each PD through an external signal.

## 4. Results and Discussion

**Figure 18:**
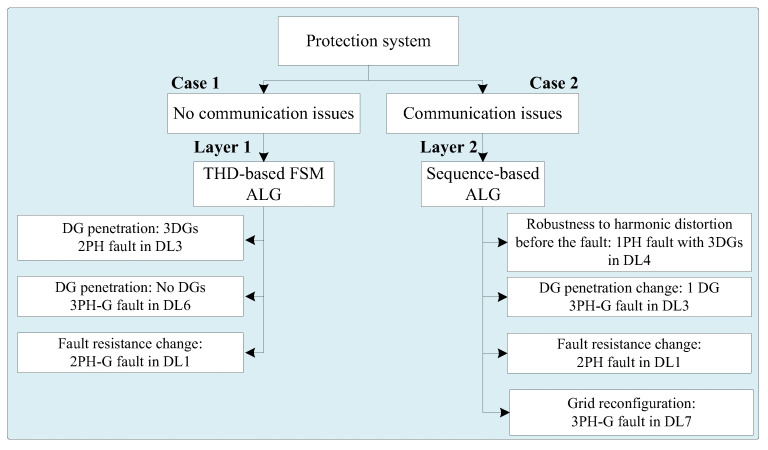
The two layers diagnose and analyze cases.

This section evaluates the dual-layer protection system using the IEEE-9 bus system in MATLAB/Simulink. The assessment covers two scenarios: first, when the system is free from communication issues, and second, when it is plagued by communication issues. Throughout the study, a range of conditions are considered, such as DG penetration, fault types, fault resistance, and fault locations. Additionally, the system’s robustness is assessed regarding harmonic distortion and its performance during grid reconfiguration. Figure 18 depicts the tested cases. The detailed parameters for the network in Figure 7 are outlined in Table 1.

### 4.1. Performance of Fault Protection in Case of No Communication Failure: Layer 1 THD-Based FSM Protection 

#### 4.1.1. Performance of Fault Protection in Case of a 2PH Fault with Three Connected DGs

**Figure 19:**
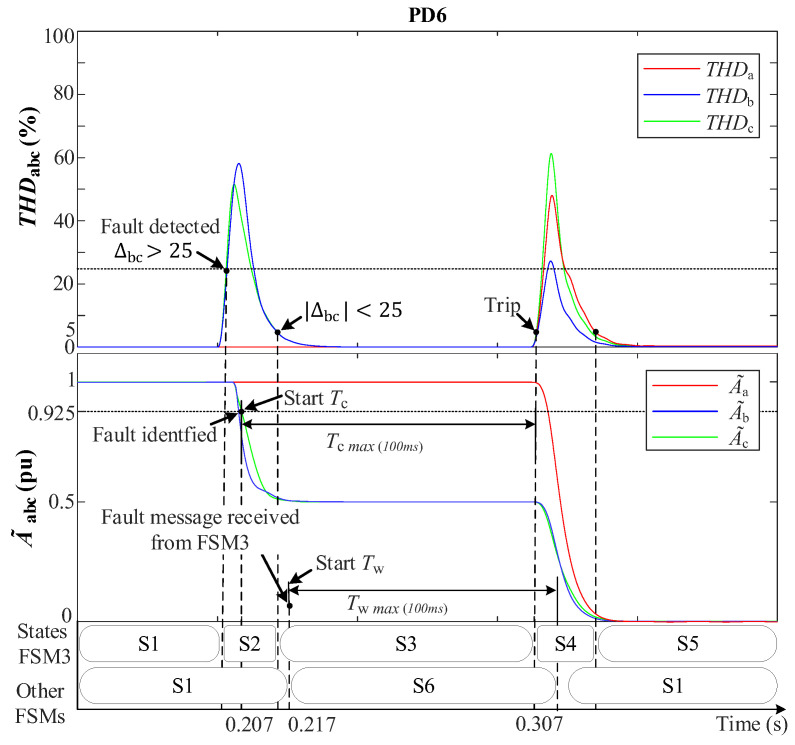
THD-based FSM fault protection behavior for a 2PH fault with 3DGs at DL3. **Upper**: ${THD}_{abc}$. **Lower**: ${\tilde{A}}_{abc}$.

In this section, a 2PH fault between phases *b* and *c* with 3DGs is examined at 0.2 s at DL3; see Figure 7. Figure 19 demonstrates the behavior of the THD-based FSM protection system during the fault.

At the time of the fault (0.2 s), there is a distinct increase only solely in ${THD}_{bc}$, while ${THD}_{a}$ remains at a steady 0%. ${\tilde{A}}_{bc}$ experiences a sharp decline to about 0.5 pu due to the fault’s impact on ground connection and fault resistance (*r* = 0.1 Ω), in contrast to the unaltered ${\tilde{A}}_{a}$*,* which maintains its value at 1 pu. Fault detection takes place precisely at 0.204 s upon the fulfillment of the ${∆}_{bc}>25$ and $\sigma_{{LPF}_{bc}}$ criteria, with recorded values stored ($\sigma_{{LPFm}_{bc}}$). 

**Figure 20:**
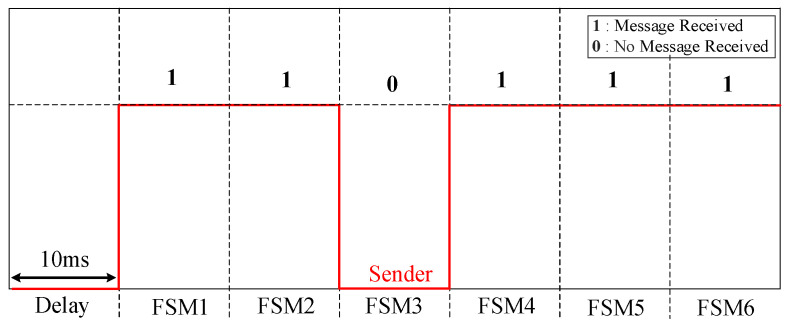
Communication signal received by each FSM.

At 0.207 s, the algorithm successfully categorizes the fault as type (BC) based on the met conditions: ${\tilde{A}}_{bc}<0.925$, ${THD}_{bc}>\sigma_{{LPFm}_{bc}}$, and $V_{abc0}=0$. Following fault detection, PD6 sends a wireless fault message to FSM3. This prompts FSM3 to transition to state S2, issue a notification to the other non-faulted FSMs, and initiate the countdown on its $T_{c}$ timer. As there are no communication impediments, the remaining FSMs receive the message after a 10 ms interval, accounting for the standard communication link delay. Consequently, they progress from S1 to S6 (hold state), simultaneously commencing the upward count on the $T_{w}$ timer. Figure 20 depicts the communication signal reception at each FSM receiver. Notably, FSM3, being the originator, does not receive the fault message.

The behavior of the ${THD}_{abc}$ signal in FSM3 exhibits a transient peak that rapidly decays to zero. Subsequently, when the condition $\left|\alpha_{bc}\right|<\alpha_{o}$ while ${\tilde{A}}_{bc}<0.925$ pu still holds, FSM3 transitions from state S2 to S3 and the timer $T_{c}$ continues to increment. Once $T_{c}$ reaches 100 ms, indicating that the fault is considered permanent, FSM3 proceeds to the fault isolation state (S4) and triggers a signal to trip the circuit breakers in DL3. The timer $T_{c}$ is reset and FSM3 advances to state S5, where it awaits reconnection instructions. While FSM3 is in state S4, a sudden spike is observed in all THD signals due to the disconnection of DL3, causing an abrupt change in grid voltages. However, this does not affect the operation of FSM3. In the non-faulted FSMs, the timer $T_{w}$ continues to increment, and upon reaching 100 ms, these FSMs return to the normal operational state (S1).

**Figure 21:**
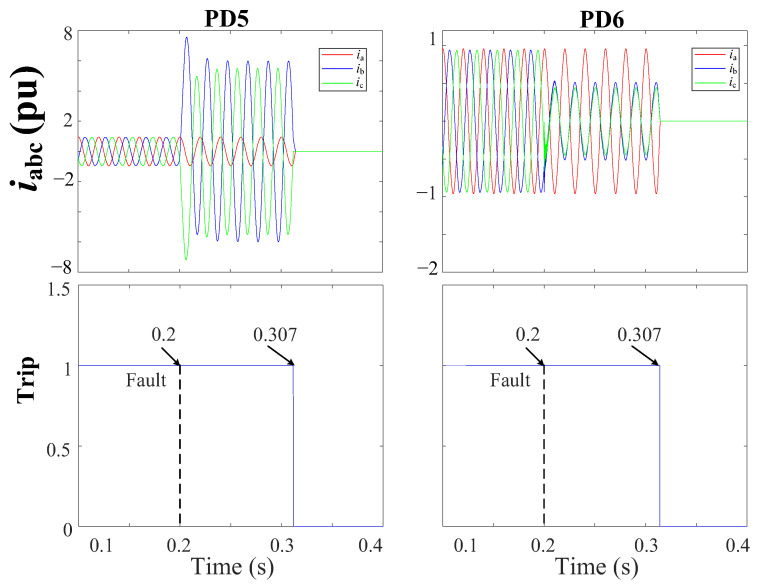
$i_{abc}$ currents and trip signals for the grid during a 2PH fault with 3DGs at DL3 using a THD-based FSM protection approach.

Figure 21 depicts the grid currents ($i_{abc}$) during a 2PH fault with 3DGs at DL3. Notably, both PD5 and PD6 of DL3 are tripped at 0.307 s in response to the tripping signal sent by FSM3.

#### 4.1.2. Performance of Fault Protection in Case of Changing DG Penetration

In this section, a 3PH-G fault is examined at 0.2 s at DL6; see Figure 7. When the DGs at Buses 7, 5, and 6 are disconnected, no power is injected into the grid. In this case, PD11 is the nearest to the fault; therefore, it is the fastest to detect the fault at 0.206 s and sends a fault message to FSM6. Then, FSM6 starts its process similarly to the previous case; $T_{c}$ starts counting up and sends another fault message to the other FSMs (non-faulted) advising them about the fault to allow them to stop their process (hold state), and $T_{w}$ starts counting up to wait for the fault to be isolated. In this case, the fault messages have been checked at these FSMs to be received without communication issues and within the normal delay. Once reaching $T_{c}=100\;ms$, the fault is declared permanent, and FSM6 trips the CBs in DL6 and isolates the fault. In non-faulted FSMs, once $T_{w}$ reaches 100 ms, they return to S1 (normal operation).

**Figure 22:**
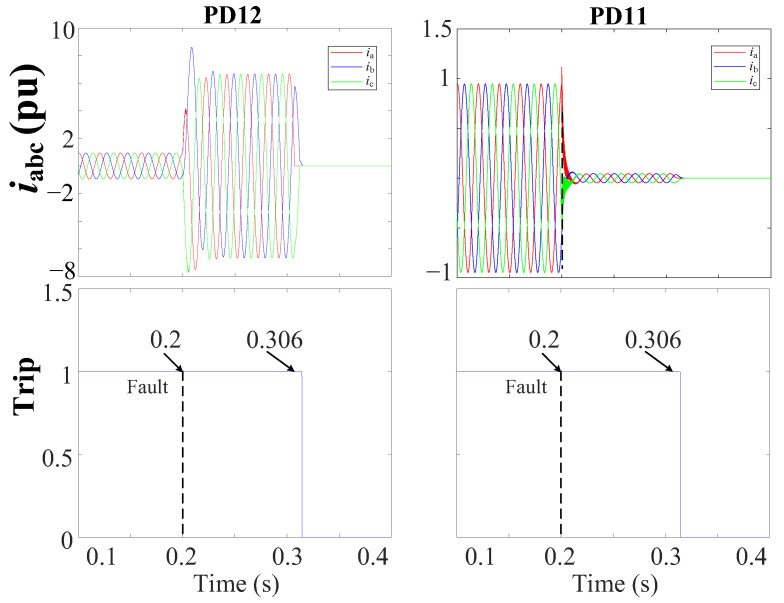
$i_{abc}$ currents and trip signals for the grid during a 3PH-G fault without DGs at DL6 using a THD-based FSM protection approach.

Figure 22 depicts the grid currents ($i_{abc}$) during a 3PH-G fault without DGs at DL6. Note that, in response to the tripping signal sent by FSM6, the circuit breakers inside both PD11 and PD12 of DL6 are tripped at 0.306 s.

The simulation results show that the changes in DG penetration have a negligible impact on the operational efficacy of the proposed protection method. In both instances, the method adeptly detects and isolates the fault efficiently.

#### 4.1.3. Performance of Fault Protection in Case of Changing the Fault Resistance

**Figure 23:**
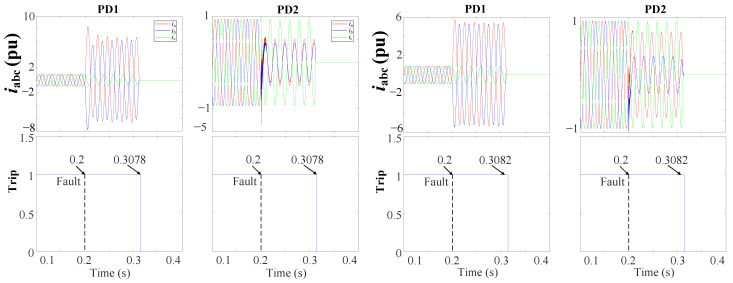
$i_{abc}$ currents and trip signals for the grid during a 2PH-G fault with 3DGs at DL1 using a THD-based FSM protection approach.

Changing the fault impedance introduces a considerable challenge in fault detection within distribution networks, posing a significant impact on the performance and effectiveness of various protection strategies. Therefore, this section explores the behavior of the THD-based FSM when an unsymmetrical 2PH-G fault (ab-g) with the 3DGs occurs in DL1 at 0.2 s; see Figure 7. The protection is tested with two fault resistances: r=0.1 Ω and r=6 Ω. Figure 23 shows the performance of the THD-based FSM during the fault with the two fault resistances. It can be observed that in both cases, the approach is capable of clearing the fault at 0.3078 s and 0.3082 s, respectively.

**Figure 24:**
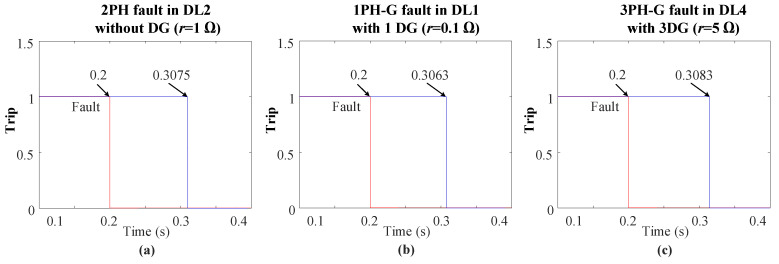
Fault clearing times of the THD-based FSM protection method during different faults types and resistances: (**a**) 2PH in DL2, (**b**) 1PH in DL1, and (**c**) 3PH-G in DL4.

Moreover, the protection method was tested under various fault resistances, types, locations, and DG penetrations. The results consistently showed that in all scenarios, the THD-based FSM has the ability to clear the fault in minimal time, as seen in Figure 24.

### 4.2. Performance of Fault Protection in Case of a Communication Failure: Layer 2-Sequence-Base Protection

#### 4.2.1. Fault Protection Robustness Test in Presence of Harmonic Distortion before the Fault

In this section, we analyze a 1PH-G fault with three DGs occurring at 0.2 s at DL4, see Figure 7. This analysis takes into account the presence of harmonic distortion prior to the fault event as well as potential communication challenges.

**Figure 25:**
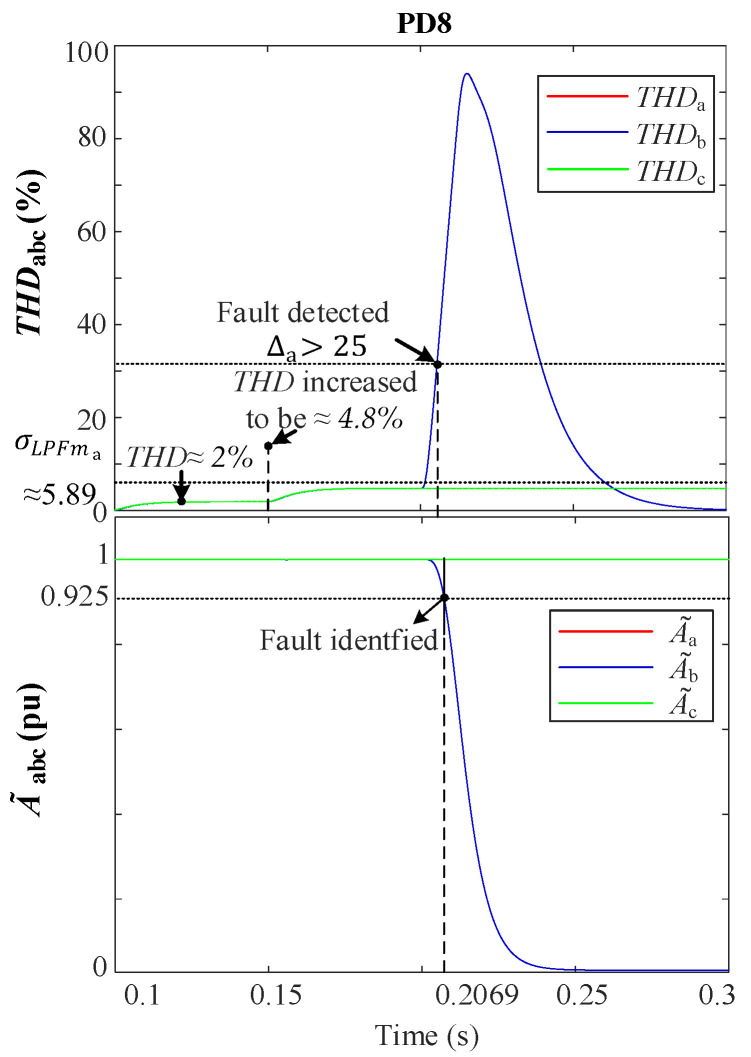
Performance of the THD fault detection during the 1PH-G fault at DL4. **Upper**: ${THD}_{abc}$. **Lower**: ${\tilde{A}}_{abc}$.

Figure 25 illustrates the THD-LPF detection algorithm’s behavior during a fault event. Prior to the fault, there was a 2% injection of the 5th harmonic at 0.1 s, which increased to 4.8% at 0.15s. At 0.2 s, a 1PH-G fault occurred in phase “b”. This caused a sharp increase in ${THD}_{b}$, while ${THD}_{ac}\;$ remained at 4.8%. The fault was detected at 0.205 s when $\alpha_{b}$ surpassed the threshold, ${∆}_{b}>\alpha_{o}$. The value of $\sigma_{{LPF}_{b}}$ was then read and stored in memory as $\sigma_{{LPFm}_{b}}$.

During the event, ${\tilde{A}}_{b}$ experiences a drop towards 0 pu due to the low fault resistance (r=0.001 Ω), whereas ${\tilde{A}}_{ac}$ remains unaffected at 1 pu. At 0.2069 s, the condition ${\tilde{A}}_{b}<0.925$ pu is satisfied, and ${THD}_{b}>\sigma_{{LPFm}_{b}}$ remains valid. Subsequently, PD8 detects the fault and classifies it as a BG fault.

**Figure 26:**
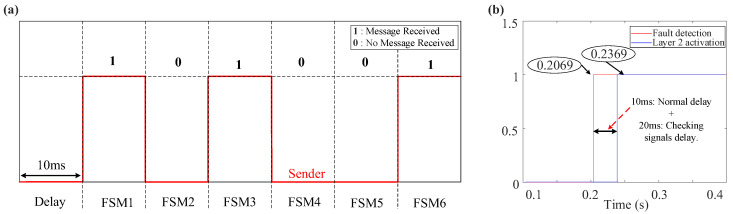
Priority system behavior during a 1PH-G fault with communication issues: (**a**) communication signals received by each FSM, (**b**) layer 2 activation signal.

At this point, the automatic activation of Layer 1 triggers FSM4 to send a fault message to non-faulted FSMs following a standard 10 ms delay. However, due to communication issues, as seen in Figure 26, FSM2 and FSM5 are assumed to remain unresponsive. Subsequently, a 20 ms delay is introduced to scrutinize the communication signals, unequivocally validating the message loss. In response, Layer 2 is engaged to execute fault isolation.

**Figure 27:**
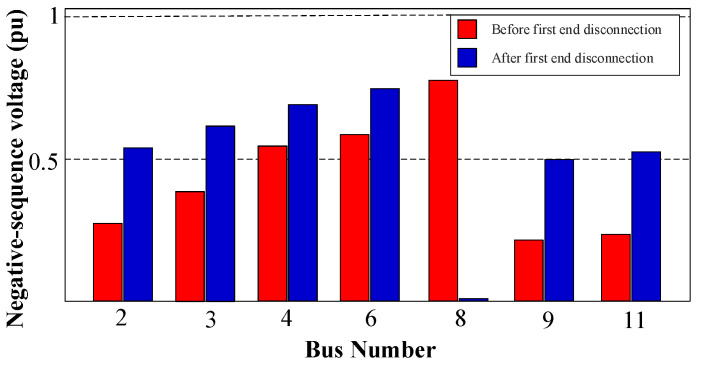
The NSV profile during a 1PH-G fault with three DGs connected before the disconnection of the first end of the faulted DL4 and after the disconnection.

Following this, sequence components are measured, and the sequence-based fault location algorithm is employed to precisely locate and isolate the fault, using the behavior of the NSV due to the fault’s unsymmetrical nature. Detailed in Figure 27 is the NSV profile of the system during the fault, wherein it is shown that bus 8 exhibits the highest NSV value. Given that only PD8 is connected to this bus, there is no necessity for applying the initial disconnecting and reclosing strategy. Consequently, the disconnection of PD8 will directly follow the characteristic curve of the definite time–voltage, as delineated in Figure 13b.

**Figure 28:**
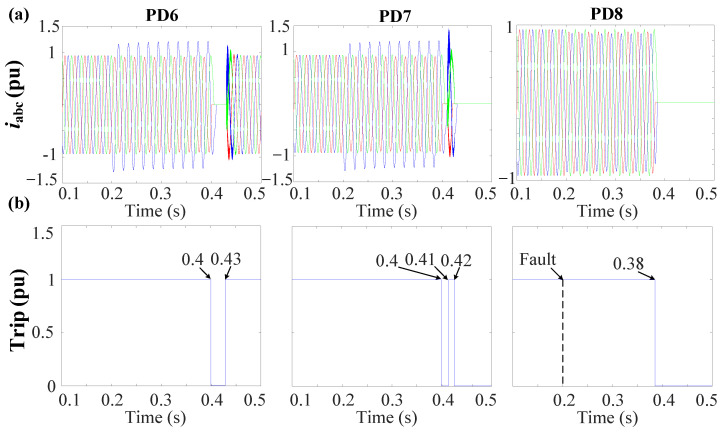
Behavior of the system during the 1PH-G fault at DL4 with 3DGs. (**a**) Grid currents ($i_{abc}$). (**b**) Trip signals.

As depicted in Figure 27, PD8, located at bus 8, exhibits the highest NSV value at 0.75 pu. With reference to Figure 13b, it can be observed that PD8 necessitates approximately t ≈ 0.18 s from the time of fault occurrence to initiate the tripping signal. Figure 28a,b provide the grid currents ($i_{abc}$) and the corresponding tripping signals, respectively, during the occurrence of the 1PH-G fault. As seen in Figure 28, PD8 initiates tripping simultaneously at 0.38 s.

To effectuate fault isolation at DL4, the identical procedure will be iterated at the second termination of the line, specifically, at bus 6 for PD6 and PD7. As shown in Figure 27, bus 6 manifests the highest NSV value subsequent to the disconnection of the first termination at DL4 (bus 8). Notably, both PD6 and PD7, being directly connected to bus 6 exhibit an equivalent NSV of 0.72 pu. Given this, Figure 13b demonstrates that the PDs will undergo tripping after an elapsed time of approximately t ≈ 0.2 s from the inception of the fault.

From Figure 28, it is apparent that PD6 and PD7 execute simultaneous tripping at 0.4 s as their initial response. Following this, both PD6 and PD7 initiate the reconnection algorithm, as shown in Figure 15. Following the first scenario, PD7 re-establishes connection after a 10 ms delay at 0.41 s. However, given that the fault persists, PD7 proceeds to detect the fault once more and subsequently triggers a trip event at 0.42 s, with this process consuming 10 ms. In contrast, PD6 necessitates 30 ms to effectuate reconnection at 0.43 s. In this particular case, as the fault has been effectively cleared, PD6 does not sense the presence of the fault and thus maintains a connected state, as seen in Figure 28.

In the event of a failed disconnection at the initial end of DL4 (bus 8), thereby leaving the CB inside PD8 in an active state, the responsibility for fault isolation shifts to PD7, located at the second end of DL4 (bus 6), as PD7 has the second-highest NSV value of 0.61 pu, as seen in Figure 27. Consequently, under such circumstances, PD7 will perform the disconnection after an approximate elapsed time of t ≈ 0.23 s from the fault.

#### 4.2.2. Performance of Fault Protection in Case of Changing DG Penetration

This section provides an evaluation of the protection system’s performance under the scenario of a symmetrical 3PH fault occurring at DL3, with one connected DG. The SOGI-THD fault detection algorithm successfully detects and identifies the fault in 6.4 ms. Following this, Layer 1 is automatically activated. However, in light of communication challenges, Layer 2 is switched on for fault isolation after a comprehensive assessment and confirmation of the communication failure occurrence.

**Figure 29:**
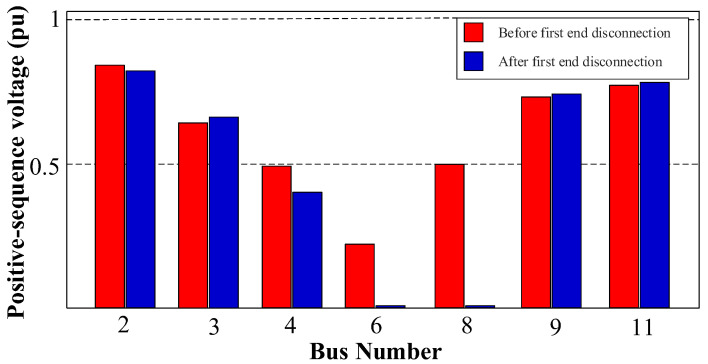
The PSV profile during a 3PH-G fault with one DG connected before the disconnection of the first end of the faulted DL3 and after the disconnection.

Following this, precise measurements of the sequence components are undertaken. Subsequently, the fault location algorithm is executed to accurately locate and isolate the fault using the behavior of the PSVs. This behavior is graphically represented in Figure 29, which offers a visual depiction of the PSV profile of the system during the fault.

Figure 29 shows that bus 6 exhibits the lowest PSV value. Both PD6 and PD7 are associated with a PSV of 0.23 pu due to their connection to bus 6. As a result, the initial action is to disconnect PD6 and PD7, following the voltage–time characteristic delineated in Figure 13. With this PSV, it is observed in Figure 13a that the PDs necessitate approximately t ≈ 0.14 s from the instance of fault occurrence to initiate a tripping signal.

**Figure 30:**
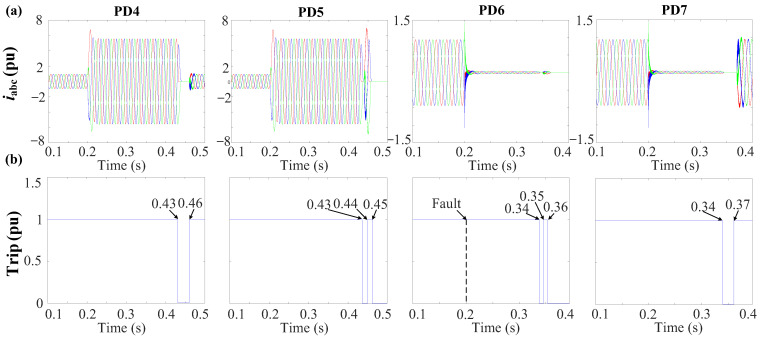
Behavior of the system during the 3PH-G fault at DL3 with 1DGs. (**a**) Grid currents ($i_{abc}$). (**b**) Trip signals.

Figure 30a,b depicts the grid currents ($i_{abc}$) and trip signals during the occurrence of a 3PH fault. From Figure 30, PD6 and PD7 undergo simultaneous tripping at 0.34 s as their initial response. Following this, both PD6 and PD7 implement the reconnection algorithm, as shown in Figure 15. Following the first scenario, PD6 successfully restores the connection after a 10 ms delay at 0.35 s. However, given the persistence of the fault, PD6 detects the fault once more and triggers a second trip event at 0.36 s. In contrast, PD7 requires 30 ms to re-establish connection at 0.37 s, and as the fault has been effectively cleared in this case, PD7 does not detect the fault’s presence and remains in a connected state, as shown in Figure 30.

To isolate the fault at DL3, the same procedure is replicated at the second end of the line, specifically at bus 4 for PD4 and PD5. As shown in Figure 29, after disconnecting the first termination at DL3 (bus 6), bus 4 exhibits the lowest PSV profile. Both PD4 and PD5 share an identical PSV value of 0.4 pu. Following Figure 13a, with these PSV values, the PDs will trip approximately t ≈ 0.23 s from the fault initiation. Figure 30 confirms that PD4 and PD5 do indeed trip at 0.43 s. Subsequently, PD5 is reconnected, then trips again as the fault persists. Following this, PD4 is reconnected. Since the fault has been successfully cleared, PD4 remains in a connected state.

The simulation results demonstrate that changing the DG penetration does not affect the performance of the protection strategy. In both scenarios, the method effectively detects and isolates the fault.

#### 4.2.3. Performance of Fault Protection in Case of Changing the Fault Resistance

**Figure 31:**
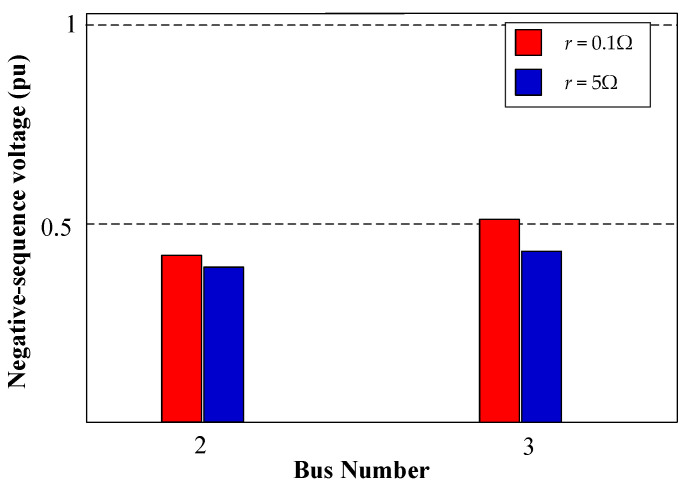
The voltage profile of negative-sequences during a 2PH fault with three DGs connected at DL1 with different fault resistances.

Changing the fault impedance can present a significant challenge for fault detection in DSs for various protection techniques. In this section, we explore different fault resistances, specifically 0.1 Ω and 5 Ω, when an unsymmetrical 2PH fault occurs at 0.2 s at DL1 (see Figure 7), while three DGs are connected. The SOGI-THD method successfully detects and identifies the fault within 7.5 ms. Subsequently, due to communication constraints, Layer 2 is activated, followed by the execution of the sequence-based fault location algorithm. Figure 31 provides the NSV profile of DL1 (Bus 2 and 3) during the fault.

**Figure 32:**
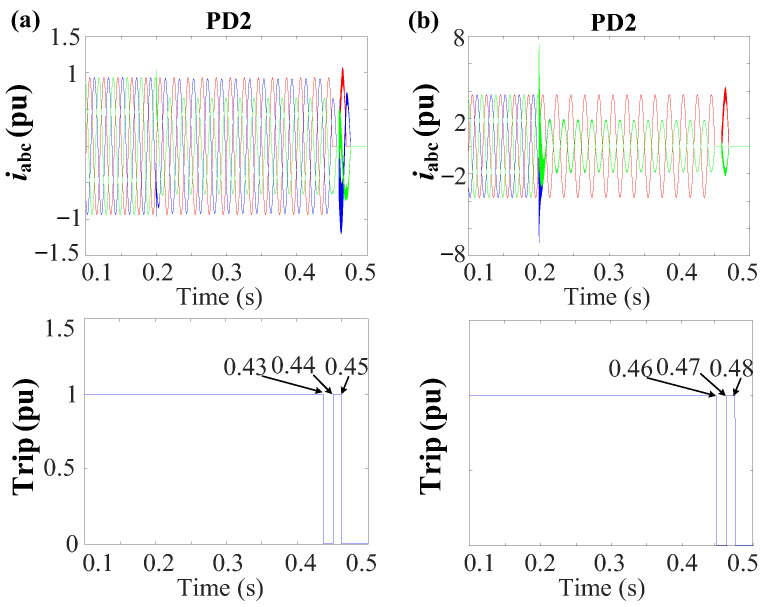
Grid currents ($i_{abc}$) and the trip signal during the 2PH fault at DL1 for various fault resistances. (**a**) *r* = 0.1 Ω. (**b**) *r* = 5 Ω.

In the scenario with *r* = 0.1 Ω, the NSV recorded at bus 3 is 0.51 pu. Therefore, following Figure 13b, PD2 initiates a trip event at t ≈ 0.23 s from the fault occurrence, approximately at 0.43 s. In contrast, for *r* = 5 Ω, the NSV at bus 3 is 0.39 pu, resulting in PD2 tripping after a time of approximately t ≈ 0.26 s, occurring at 0.46 s. Figure 32 presents the grid currents ($i_{abc}$) and the corresponding trip signals during the 2PH fault with the examined fault resistance.

The simulation results show that variations in fault resistance directly influence the disconnection timing of PDs due to their impact on NSV. Nevertheless, this observed deviation does not undermine the robustness of the protection strategy. In both scenarios, the method consistently exhibits its proficiency in promptly detecting and effectively isolating faults.

#### 4.2.4. Performance of Fault Protection in Case of Grid Reconfiguration

**Figure 33:**
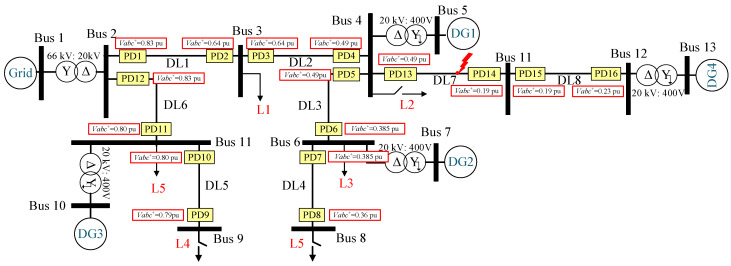
Positive-sequence voltage of each PD during the 3PH fault at DL7.

This section examines the efficacy of the protection method in the case of DS reconfiguration, emphasizing its effectiveness under these distinctive conditions. To further evaluate its capabilities, two DLs (DL7 and DL8) have been integrated into the DS through bus 4, as seen in Figure 33. Note that the parameters of these new DLs conform to those stipulated in Table 1. Subsequently, a comprehensive analysis is conducted to examine the method’s response to a symmetrical 3PH fault occurring in DL7 at 0.2 s. 

**Figure 34:**
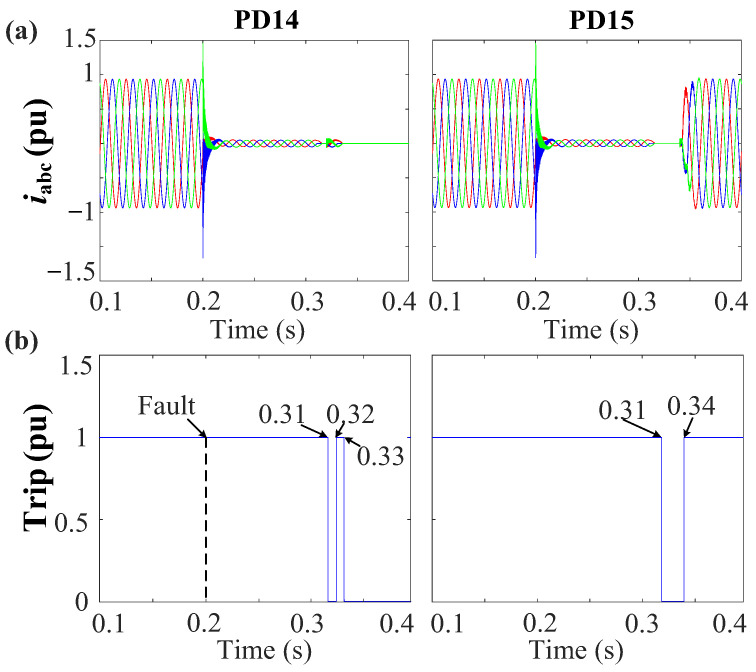
Behaviour of the system during the 3PH fault at D7 in the case of grid reconfiguration. (**a**) Grid currents ($i_{abc}$). (**b**) Trip signals.

The detection algorithm identifies the fault within approximately 6.8 ms. Subsequently, the fault location algorithm is promptly engaged to isolate the fault. Figure 33 depicts the PSV profile of each PD in response to the grid configuration change. PD14, located at bus 11, exhibits the lowest PSV value at 0.19 pu. Following Figure 13a, PD14 will execute a trip operation at t ≈ 0.11 s from the fault initiation, precisely at 0.31 s. Figure 34 shows the grid currents, $i_{abc}$, and trip signals. The simulation results show the adaptability of the protection strategy in response to the grid configuration changes.

Additionally, the protection techniques proposed in this study are compared with other methods in similar conditions. Table 2 compares the methods based on their response speed, accuracy, cost of implementation, the use of inverter-based systems, communication needs, and the grid configuration used for testing.

## 5. Experimental Verification

**Figure 35:**
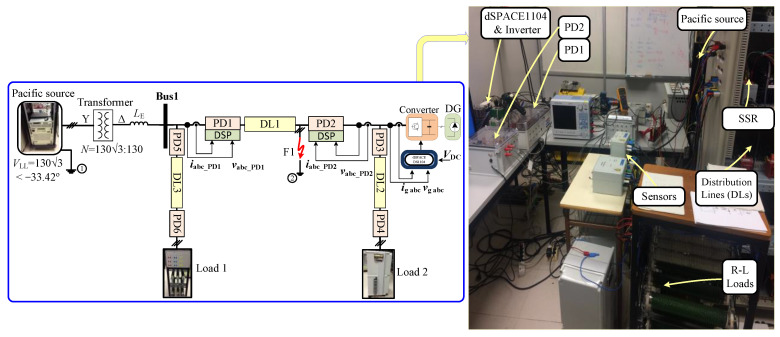
The radial grid laboratory implementation and experimental setup.

To experimentally validate the proposed algorithm, an equivalent model was constructed in the laboratory to replicate the identical response and dynamic behavior observed in the analyzed grid. This study focused on a radial grid network configuration integrated with DGs. The practical implementation of the proposed algorithm involved the utilization of a numerical relay, based on DSP, to process measurement data acquired from sensors. The relay then executed the algorithms, ultimately providing appropriate decisions in the form of trip signals. The Solid State Relay (SSR) functioned as a PD within the examined radial grid. Figure 35 depicts the detailed equipment setup of the laboratory-implemented grid, while Table 3 outlines the parameters of the devices employed in the experimental setup. Figure 35 offers an overview of the laboratory setup emulating the radial grid. It incorporates three lines (DL1, DL2, and DL3) to represent the examined grid. The adjustments include doubling the equivalent value of DL2 and tripling that of DL3 in comparison to DL1. Additionally, points 1 and 2 are connected to the common ground point, which corresponds to the ground of the Smart Source. The load was arranged in an isolated star configuration. To accurately replicate the impedance characteristics of the main grid, an inductor was introduced before bus 1, as illustrated in Figure 35.

The aim is to replicate identical voltages, currents, and impedances in per unit (pu) within the laboratory system, ensuring precise conformity with the actual operational dynamics. In the real DS grid, the designated base values are *V*_Base_ = 20 kV, *S*_Base_ = 25 MVA, and *I*_Base_ = 721.69 A, detailed in Table 4. These specific base values are intentionally chosen to establish per unit equivalency in the voltage and current during the steady-state conditions of a radial grid. The strategic choice of laboratory base values is designed to facilitate experimentation with reduced voltages and currents while maintaining per unit results consistent with those observed in the authentic grid.

The algorithms are executed using a DSP TMS320F28335. Additionally, ControlDesk and dSPACETM DS1104 are utilized for implementing the inverter control. Both the algorithms and inverter control are programmed using Matlab^TM^ software 2023.a. Various symmetrical and unsymmetrical faults underwent rigorous testing to assess the efficacy of the protection system. In this phase, validation was prompted by a communication anomaly, leading to the activation of the second layer (sequence-based). This evaluation specifically targeted a single-phase fault occurrence at 0.5 s, specifically in the F1 fault location, illustrated in Figure 35.

**Figure 36:**
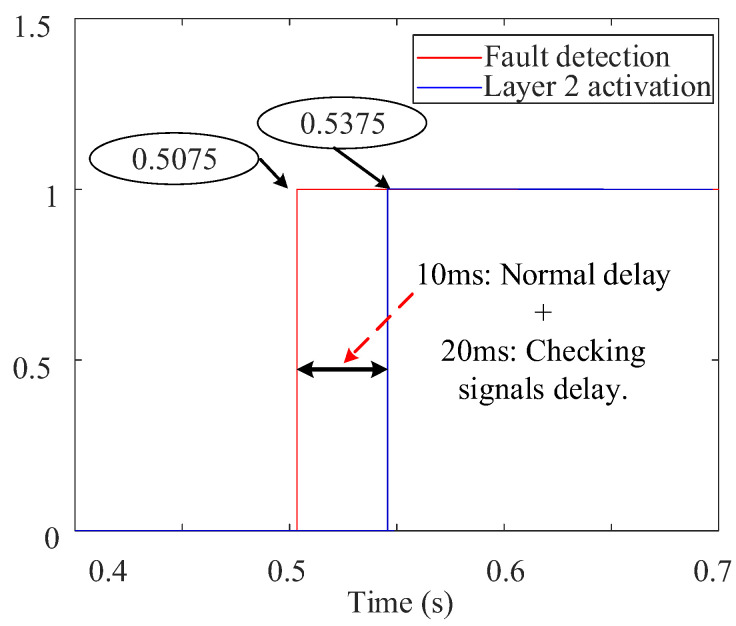
Priority system behavior during a 1PH-G fault with communication issues.

In Figure 36, the first layer is activated automatically, detecting the fault in 7.5 ms. Due to communication issues, a given delay is introduced for checking and confirming the failure. Subsequently, Layer 2 is engaged to isolate the fault.

Following this, the sequence components are measured, and the sequence-based fault location layer is employed to precisely locate and isolate the fault, using the behavior of the NSV due to the fault’s unsymmetrical nature. The NSV profile of the system during the fault depicts that the bus situated at the terminal of the faulted line (DL1) and connected to PD2 and PD3 exhibits the highest NSV value of 0.82 pu. With reference to the definite time–voltage curve outlined in Figure 13b and employing a procedure akin to that in Figure 11, it can be observed that PD8 necessitates approximately t ≈ 0.15 s from the time of fault occurrence to initiate the tripping signal.

**Figure 37:**
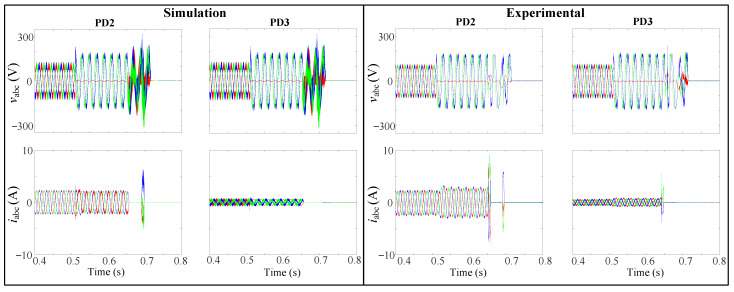
Simulation and experimental behaviour of $v_{abc}$ and $i_{abc}$ during the 1PH-G fault at DL1.

Figure 37 illustrates the simulation and the experimental behavior of the grid voltages, $v_{abc}$, grid currents, $i_{abc}$, and the corresponding tripping signals during the occurrence of the 1PH-G fault, respectively. As seen in Figure 37, both PD2 initiates tripping simultaneously at 0.65 s.

Following this, both PD2 and PD3 implement the reconnection algorithm. Following the first scenario, PD3 successfully restores the connection after a 10 ms delay at 0.66 s. However, given the radial system configuration and PD2’s ongoing disconnection, it will remain unaffected by the fault. In contrast, PD2 requires 30 ms to re-establish connection at 0.7 s, and promptly trips again as the fault is not cleared. To isolate the fault at DL1, the same procedure is replicated at the second end of the line, specifically for PD1 and PD5. The agreement observed between the experimental and simulated outcomes, as seen in Figure 37, provides validation for the efficiency of the proposed methodology. It is essential to emphasize that the SSR behaves differently than the ideal breaker. In Figure 37, the substantial overcurrent in the experimental $i_{abc}$ currents of PD2 and PD3 deviates from the simulated results. This discrepancy is primarily attributed to the SSRs, which open instantly, generating sparks and leading to a significant increase in current, nearly doubling the expected levels.

## 6. Conclusions

This paper introduces a dual-layer protection system designed to mitigate fault events within DSs, providing rapid and reliable fault protection even in the event of potential communication failures. The first layer incorporates the THD, the estimated amplitude voltages, and the zero-sequence grid voltage components to formulate a protection strategy. This strategy is implemented through an FSM for the purpose of fault detection and isolation within the grid. The effectiveness of this layer is contingent on robust communication protocols for effective coordination.

The second layer of the protection system capitalizes on the distinctive behavior of PSV and NSV during fault events, enabling precise fault localization and isolation. This layer operates autonomously, utilizing localized data from PDs without relying on communication channels to transmit trip signals. The SOGI-FLL is used for the derivation of the estimated variables and sequence components, ensuring fast detection with minimal computational overhead.

A priority system is defined to manage the interactions between the two protection layers, offering an effective solution in cases of communication disruption. This prioritization ensures the highest level of security and reliability, thereby enhancing system redundancy. Specifically, upon detecting a fault, the first layer is automatically activated, and the communication signal is checked to verify the availability of the detection decision. In the event of a loss of communication signals, a secondary verification is performed to definitively rule out any transient issues. If the signals are received, the first layer continues its operation to isolate the fault. However, in cases where it is confirmed that communication signals are permanently lost even after re-evaluation, the second layer serves as a secondary protection at each PD to isolate the fault.

The system was tested for various fault types, including situations with a high penetration of DG and both low and high fault resistances. Across all test cases, the simulation and experimental results validate the efficacy of the protection system, demonstrating its prompt and reliable response to faults, even in the presence of potential communication disruptions. The fault detection times consistently fell within the range of 6 to 8.5 ms. Furthermore, the system works with minimal computational overhead and is adaptable to grid reconfiguration, which not only enhances stability, but also fortifies redundancy in the system.

In subsequent studies, we envisage delving into the impact of high-voltage grid codes on our protection scheme, specifically assessing their adaptability and efficacy under varying transformer configurations. This exploration aims to enhance the comprehensiveness of our findings and contribute valuable insights to the field of power system protection. We recognize the importance of extending our research to encompass a broader range of grid scenarios, and future studies will be dedicated to addressing this aspect to ensure a more comprehensive understanding of the protection system’s performance across diverse grid environments.

Moreover, another future research endeavor can address critical gaps identified in this study. Firstly, an exploration of the frequency and economic impact of communication loss between protective devices in modern distribution grids will be undertaken, shedding light on the practical implications and potential economic consequences of such disruptions. Additionally, experimental validation will be extended beyond laboratory emulations to encompass real-world distribution networks with DERs. This expansion aims to assess the effectiveness of the proposed solution in practical deployment scenarios, specifically in modern dispatch centers of Distribution System Operators (DSOs) and SCADA systems. This multifaceted approach will enhance the robustness and applicability of the proposed solution in addressing protection challenges in real-world distribution environments.

## Figures and Tables

**Table 1:** Parameters of the System.

Main Grid	HV/MV Transformer (YNd11)	Distribution Line (DL)	MV/LV Transformer (Dyn11)	DGs Rating
Rated voltage: 66 kV Short circuit power: 360 MVA	Rated power: 20 MVA Rated Voltage: 66/20 kV $U_{SC}$(%): 11	Resistance: 0.16 Ω/km Reactance: 0.109 H/km Capacitance: 0.31 μF/km Length of line: 2 km	Rated power: 400 kVA Rated Voltage: 20/0.4 kV $U_{SC}$(%): 4.5	Rated power: 6 MVA Rated Voltage: 400 V

**Table 2:** Comparison of different protection approaches operating under similar conditions.

References	Protection Strategies	Parameter Used	Speed	Accuracy	Cost	Inverter-Based	Communication	Grid Configuration
[17]	Wavelet individual entropy and fuzzy inference system	Current	High	High	Low	Yes	Not Required	Radial
[20]	fuzzy logic technique	Current	High	Medium	Low	No	Not Required	Radial/Ring
[19]	Wavelet transform and support vector mechanism	Voltage	Medium	High	High	No	Not Required	Radial/Ring
[21]	Statistical morphology, recursive least square methods, and Butterworth filter	Current	High	High	High	Yes	Required	Ring
[23]	Differential phase angle criteria	Voltage	High	High	High	Yes	Required	Ring
[24]	S-transform	Current and Voltage	Medium	Medium	High	Yes	Required	Radial
[25]	Power spectral density and transform.	Current	High	High	High	Yes	Not Required	Radial
[26]	Deep belief network, time–time transform and PUM	Current	Low	High	High	Yes	Required	Radial/Ring
[18]	Travelling wave and wavelet analysis	Current	High	High	High	No	Required	Radial
[22]	Least square Adaline algorithm and modified support vector mechanism	Current	Medium	High	High	Yes	Not Required	Radial/Ring
[27]	Hilbert–Huang Transform differential relay	Current	High	High	High	Yes	Required	Ring
[proposed approach]	SOGI-THD	Voltage	High	High	Low	Yes	Required	Radial
[proposed approach]	Sequence components	Voltage	Medium	High	High	Yes	Not required	Ring/Radial

**Table 3:** List of grid components and their parameters.

CINERGIA^TM^ Inverter	Rated power	10 kVA
	Rated voltage	400 V
Filter	Inductance (*L*)	10 mH
	Resistance (*R*)	0.2 Ω
DC bus	DC rated voltage	800 V
Three-phase Smart Power Source	345 AMXT	4.5 kVA
Three-phase power transformer	Rated power Rated L-L voltages	7.5 kVA 380/220 V
Inductor to emulate the grid	*L*_E_	10.94 mH ≈ 11 mH
Fault resistance per phase	*R*	R = 2.3 Ω
Distribution Lines (DL1, DL2, DL3)	Inductance (*L*_1_, *L*_2_, *L*_3_)	1.37, 2.74, 4.11 mH
	Resistance (*R*_1_, *R*_2_, *R*_3_)	625, 1250, 1875 mΩ
	Capacitance (*C*_1_, *C*_2_, *C*_3_)	0.316, 0.632, 10, 30 µF
Loads	Inductance (*L*_1_)	10, 30 mH
	Resistance (*R*_1_)	14.5, 42 Ω

**Table 4:** Comparison between the real grid and the laboratory grid parameters.

	Analyzed Grid	Laboratory Grid
Base voltage	*V*_Base_ = 20 kV	*V*_Base_ = 75 V
Base apparent power	*S*_Base_ = 25 MVA	*S*_Base_ = 540 W
Base current	*I*_Base rms_ = 721.69 A	*I*_Base rms_ = 2.4 A
Base impedance	*Z*_Base_ = 16 Ω	*Z*_Base_ = 31.25 Ω
Line resistance	*R*_Line_pu_ = 0.02 pu	*R*_Line_pu_ = 0.02 pu
Line inductance	*X*_L Line_pu_ = *j*0.0136 pu	*X*_L Line_pu_ = *j*0.0138 pu
Fault resistance	*R*_fault_pu_ = 0.0746 pu	*R*_fault_pu_ = 0.0746 pu

## Data Availability

Data are contained within the article.
